# Structural Characterization and Ligand/Inhibitor Identification Provide Functional Insights into the *Mycobacterium tuberculosis* Cytochrome P450 CYP126A1*

**DOI:** 10.1074/jbc.M116.748822

**Published:** 2016-12-08

**Authors:** Jude T. Chenge, Le Van Duyet, Shalini Swami, Kirsty J. McLean, Madeline E. Kavanagh, Anthony G. Coyne, Stephen E. J. Rigby, Myles R. Cheesman, Hazel M. Girvan, Colin W. Levy, Bernd Rupp, Jens P. von Kries, Chris Abell, David Leys, Andrew W. Munro

**Affiliations:** From the ‡ Manchester Institute of Biotechnology, School of Chemistry, University of Manchester, 131 Princess Street, Manchester M1 7DN, United Kingdom,; the § Department of Chemistry, University of Cambridge, Lensfield Road, Cambridge CB2 1EW, United Kingdom,; the ¶ School of Chemistry, University of East Anglia, Norwich Research Park, Norwich NR4 7TJ, United Kingdom, and; the ‖ Leibniz-Institut für Molekulare Pharmakologie, Robert-Rössle-Strasse 10, 13125 Berlin, Germany

**Keywords:** cytochrome P450, electron paramagnetic resonance (EPR), enzyme structure, high throughput screening (HTS), mass spectrometry (MS), Mycobacterium tuberculosis, CYP126A1, redox potentiometry

## Abstract

The *Mycobacterium tuberculosis* H37Rv genome encodes 20 cytochromes P450, including P450s crucial to infection and bacterial viability. Many *M. tuberculosis* P450s remain uncharacterized, suggesting that their further analysis may provide new insights into *M. tuberculosis* metabolic processes and new targets for drug discovery. CYP126A1 is representative of a P450 family widely distributed in mycobacteria and other bacteria. Here we explore the biochemical and structural properties of CYP126A1, including its interactions with new chemical ligands. A survey of azole antifungal drugs showed that CYP126A1 is inhibited strongly by azoles containing an imidazole ring but not by those tested containing a triazole ring. To further explore the molecular preferences of CYP126A1 and search for probes of enzyme function, we conducted a high throughput screen. Compounds containing three or more ring structures dominated the screening hits, including nitroaromatic compounds that induce substrate-like shifts in the heme spectrum of CYP126A1. Spectroelectrochemical measurements revealed a 155-mV increase in heme iron potential when bound to one of the newly identified nitroaromatic drugs. CYP126A1 dimers were observed in crystal structures of ligand-free CYP126A1 and for CYP126A1 bound to compounds discovered in the screen. However, ketoconazole binds in an orientation that disrupts the BC-loop regions at the P450 dimer interface and results in a CYP126A1 monomeric crystal form. Structural data also reveal that nitroaromatic ligands “moonlight” as substrates by displacing the CYP126A1 distal water but inhibit enzyme activity. The relatively polar active site of CYP126A1 distinguishes it from its most closely related sterol-binding P450s in *M. tuberculosis*, suggesting that further investigations will reveal its diverse substrate selectivity.

## Introduction

The human pathogen *Mycobacterium tuberculosis* remains a major global cause of mortality as the infectious bacterium that causes tuberculosis (TB)^8^ (1). Recent data from the World Health Organization indicate that TB is the leading cause of human death worldwide among infectious diseases (2). The mortality rate in TB victims may be increased by co-infection with the human immunodeficiency virus (HIV). Moreover, the development of *M. tuberculosis* strains resistant to leading drugs usually results in extended treatment times (2). Multidrug-resistant (MDR) and extensively drug-resistant *M. tuberculosis* strains are resistant to at least the two leading TB drugs (rifampicin and isoniazid) or to both of these drugs as well as to any one of the quinolone drugs and to at least one of the second-line injectable TB drugs amikacin, capreomycin, and kanamycin (3, 4). Consequently, there is increased need for development of new TB drugs with novel modes of action. This need has been partially met recently by the development of drugs such as delamanid (which inhibits cell wall mycolic acid synthesis) and bedaquiline (an *M. tuberculosis* ATPase proton pump inhibitor), both of which have been authorized for use in MDR TB treatment (5).

A revelation from the first genome sequence of *M. tuberculosis* (that for the virulent H37Rv strain) was that 20 different cytochrome P450 (CYP or P450) enzymes were encoded (1). This large number of P450s suggested important functions for these enzymes, and key roles for *M. tuberculosis* P450s were identified in the metabolism of host cholesterol/cholest-4-en-3-one (CYP125A1 and CYP142A1) and branched chain lipids (CYP124A1), oxidative tailoring of cyclic dipeptides (CYP121A1), hydroxylation of menaquinone (CYP128A1), and sterol demethylation (CYP51B1) (6–14). The *CYP125A1* and *CYP142A1*genes are located in a gene regulon associated with host cholesterol metabolism and uptake and are required for oxidative metabolism of the steroid side chain to initiate its catabolism by *M. tuberculosis* in the macrophage (7, 8, 15). CYP128A1 is implicated in the synthesis of a virulence-associated sulfolipid (S881) through hydroxylating menaquinone 9, (MK9H_2_), the sole quinol electron carrier in the *M. tuberculosis* respiratory chain. CYP128A1 catalyzes terminal hydroxylation of MK9H_2_ to enable sulfation at the hydroxyl group by the sulfotransferase Stf3 encoded by the gene *Rv2267c*, immediately downstream of *CYP128A1* (1, 12).

The first *M. tuberculosis* P450 to be structurally and biochemically characterized was CYP51B1, the first member of the *CYP51* (sterol demethylase) gene family identified in a prokaryote (13, 16, 17). The CYP51B1 Fe^II^-CO complex is unstable and collapses from the cysteine thiolate-coordinated P450 form to the thiol-coordinated P420 state. However, the thiolate-coordinated form is stabilized by binding of estriol (14). Later studies on the *M. tuberculosis* cholesterol hydroxylase CYP142A1 and the *Sorangium cellulosum* epothilone C/D epoxidase EpoK showed that binding of substrates (cholest-4-en-3-one and epothilone D, respectively) regenerated the P450 state when added to the Fe^II^-CO P420 forms (8, 18). Importantly, the soluble CYP51B1 enzyme catalyzes oxidative 14α-demethylation of lanosterol, 24,25-dihydrolanosterol, and the plant sterol obtusifoliol and also binds azole drugs used clinically to inhibit fungal CYP51 enzymes (13, 17). These findings inspired research to examine the potency of azole drugs against mycobacteria. *In vitro* studies revealed that several azoles had good MIC values against *Mycobacterium smegmatis*, particularly econazole (<0.1 μg/ml) and miconazole (0.1 μg/ml). The same two azoles were also the most effective against *M. tuberculosis* H37Rv, albeit with higher MIC values (8 μg/ml for both drugs) (19, 20). This is possibly due to lower azole penetration into *M. tuberculosis* cells or to drug efflux (21). Studies in mice also showed that econazole reduced bacterial burden by 90% in lungs and spleen and was also effective against MDR *M. tuberculosis* strains (22, 23). Thus, regardless of issues surrounding cross-reactivity of azole drugs with human P450s, various azoles are clearly potent inhibitors of *M. tuberculosis* P450s and are important tools for characterization of these enzymes (13, 24).

Several of the *M. tuberculosis* P450s remain structurally uncharacterized. Among these is CYP126A1, a P450 with ∼35% amino acid identity to the cholesterol-oxidizing *M. tuberculosis* CYP142A1 and CYP125A1. The *CYP126A1* (*Rv0778*) gene is located in a region close to genes encoding the sterol demethylase P450 CYP51B1 (*Rv0764c*) and the uncharacterized CYP123A1 (*Rv0766c*), an enzyme that is predicted to be associated with the *M. tuberculosis* H37Rv cell membrane by 2D LC-MS analysis (25). *CYP126A1* is also located between genes involved in purine synthesis (*purB*, *Rv0777*; *purC*, *Rv0780*; and *purD*, *Rv0772*), although there is no evidence for its involvement in this pathway. However, *CYP126A1* is highly conserved across pathogenic and non-pathogenic *Mycobacterium* species, suggesting an important evolutionary role for the protein.

In previous studies, we have taken a fragment-based approach to identify novel small molecule ligands for *M. tuberculosis* P450s, including CYP121A1, CYP125A1, and CYP126A1 (26–28). In this paper, we present detailed biochemical, spectroscopic, and structural data for CYP126A1, including studies of its binding to novel substrates and inhibitors identified from a library of ∼40,000 compounds structurally related to the World Drug Index (29). The crystal structures of CYP126A1 in complex with the azole drug ketoconazole and with inhibitor and substrate-like molecules from the screen are presented, illustrating the capacity of CYP126A1 to bind bulky compounds with several rings. Native CYP126A1 was found to form a crystallographic dimer in which both open and closed forms of the protein (with respect to P450 conformation and active site access) are observed. In contrast, the CYP126A1-ketoconazole structure is a crystallographic monomer. These data provide important insights into the structural properties and the molecular selectivity of this *M. tuberculosis* P450 enzyme, highlighting its capacity to bind large compounds, and the ability of molecules with nitroaromatic groups to “moonlight” as substrates through inducing formation of high spin (HS) heme iron in CYP126A1. This is the first report of structural data for the widely conserved *M. tuberculosis* CYP126A1 P450 enzyme and provides important insights into CYP126A1 molecular selectivity and structural adaptation to the binding of large compounds.

## Results

### 

#### 

##### Expression and Purification of CYP126A1

**FIGURE 1.**
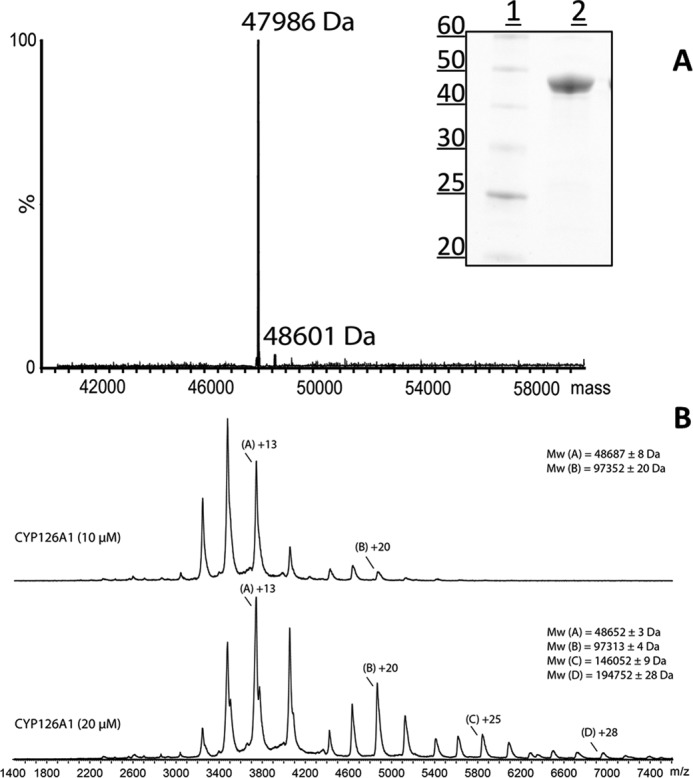
**Purification and mass spectrometry of CYP126A1.** *A*, the *main image* shows the native mass spectrum of CYP126A1. The major feature at 47,986 Da is consistent with the predicted mass of the His-tagged CYP126A1 apoprotein following removal of the initiator methionine (47,986.12 Da). The minor feature (48,601 Da) is consistent with the mass for the heme-bound CYP126A1 holoprotein. The *inset* shows an SDS-polyacrylamide gel with molecular mass markers (sizes in kDa) in *lane 1* and a sample of purified CYP126A1 in *lane 2. B*, nano-ESI native mass spectra data of ligand-free CYP126A1 at concentrations of 10 μm (*top spectrum*) and 20 μm (*bottom spectrum*). For the 10 μm sample, differently charged species are *labeled A* (for monomer) and *B* (for a minor dimer species). For the 20 μm sample, species are *labeled A–D* for the monomer through tetramer forms. Apparent masses of these different species are indicated.

CYP126A1 was purified from *Escherichia coli* expression cell extracts using Ni-IDA chromatography followed by ion exchange chromatography using Q-Sepharose. Using the Reinheitszahl (Rz) (*A*_418_/*A*_280_) value as a guide to CYP126A1 protein purity, it was found that a typical Ni-IDA step resulted in an ∼45–50-fold purification, with a further 3–4-fold purification achieved using Q-Sepharose ion exchange chromatography. At this stage, CYP126A1 was analyzed by SDS-PAGE and found to be highly purified, with an apparent molecular mass of ∼47 kDa (Fig. 1*A*, *inset*). For protein destined for crystallography and P450 structural studies, either a second Q-Sepharose purification step under the same conditions or a gel filtration step using Sephacryl S-200 size exclusion chromatography (both on an AKTA purifier) was used to ensure near homogeneity of the CYP126A1 sample.

MS analysis of purified CYP126A1 produced a mass of 47,986 Da, which is consistent with that predicted for the His_6_-tagged form of the heme-free apoprotein, after removal of the N-terminal methionine (calculated mass = 47,986.12 Da). A small component with a mass of 48,601 Da is probably due to the heme-bound form of CYP126A1 (calculated mass ∼48,602.62 Da) (Fig. 1*A*). Analysis of CYP126A1 by nano-electrospray ionization (nano-ESI) native mass spectrometry revealed the enzyme to be predominantly monomeric (Fig. 1*B*) at a relatively low concentration (10 μm). Peaks in the region *m/z* 3000–4200 were assigned to the CYP126A1 monomer and were calculated to have a mass of 48,687 Da. A small amount of dimeric protein was also present in the region *m*/*z* 4300–5300, with a mass of 97,352 Da. Analysis of CYP126A1 at a higher concentration (20 μm) indicated a propensity for the protein to self-associate, forming small amounts of trimeric (*m*/*z* 5300–6200, mass 14,6052 Da) and tetrameric (*m*/*z* 6200–7300, mass 194,752 Da) oligomers, in addition to the CYP126A1 monomer and dimer. Small deviations in masses probably arise from insufficient evaporation of buffer components under the soft ionizing conditions used (30).

##### UV-visible Spectroscopic Properties of CYP126A1 and Its Complexes with Azole Drugs

**FIGURE 2.**
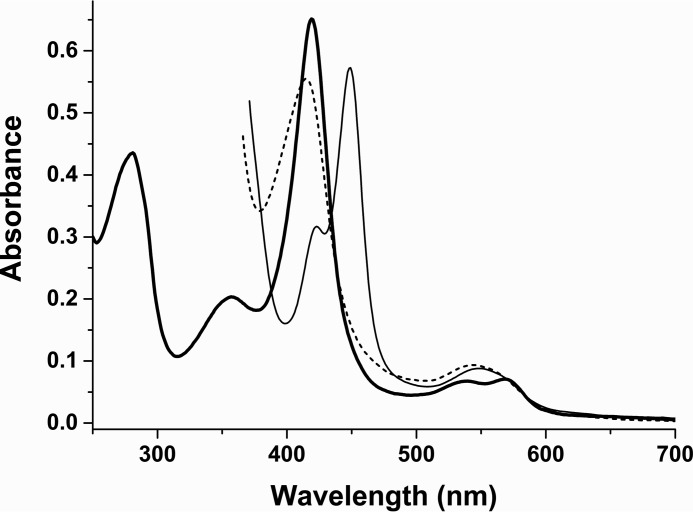
**UV-visible spectral properties of CYP126A1.** The ferric form of CYP126A1 (6.5 μm) has absorbance band maxima at 418.5 nm (Soret), 568.5 nm (α) and 539 nm (β) (*thick solid line*). Reduction with sodium dithionite results in a Soret shift to ∼412 nm with a Q-band feature at ∼544 nm (*dashed line*). Binding of carbon monoxide results in a Soret shift to 448.5 nm with a Q-band feature at ∼549 nm (*thin solid line*), consistent with retention of cysteine thiolate coordination. Over a period of ∼10–15 min, this P450 form converts to a P420 (thiol-coordinated) form with a Soret maximum at 423 nm.

**FIGURE 3.**
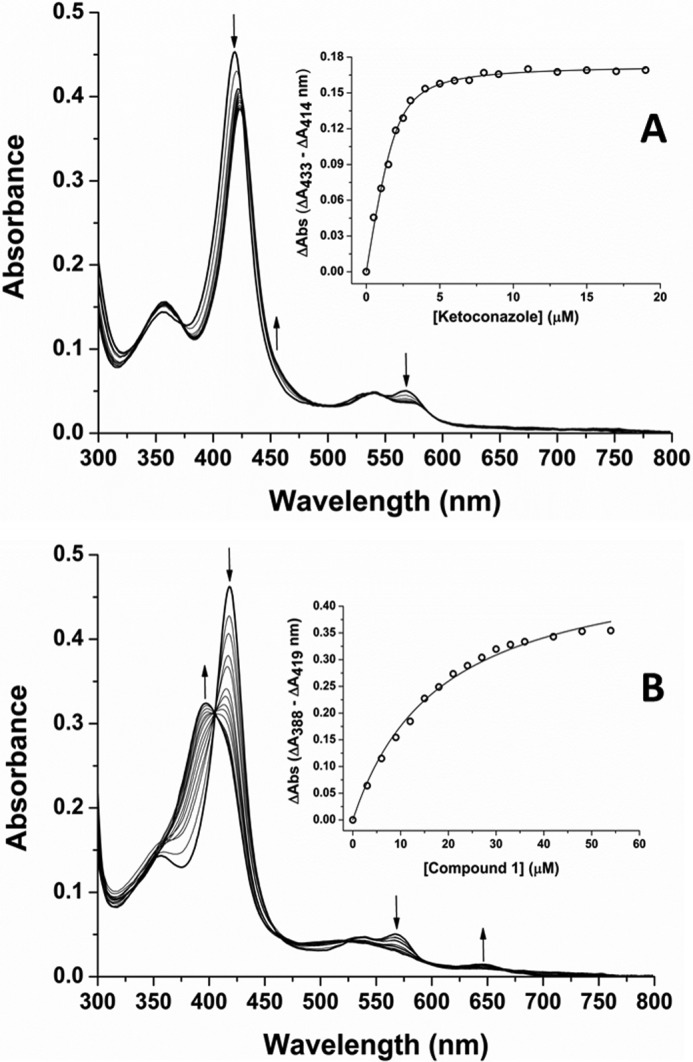
**Binding of the azole inhibitor ketoconazole and HTS compound 1 to CYP126A1.** *A*, a CYP126A1 (4.5 μm) titration with the azole drug ketoconazole. Ketoconazole coordinates the P450 ferric heme iron and induces a type II red shift of the heme Soret band from 418.5 to ∼424 nm at near saturation. Isosbestic points are located at ∼373, 502, and 549 nm. The *inset* shows a plot of the maximal ketoconazole-induced heme absorption change (Δ*A*_433_ − Δ*A*_414_) *versus* ligand concentration, with data fitted using a tightly binding (Morrison) quadratic function to give a *K_d_* of 0.34 ± 0.07 μm (65). In both *panels*, *arrows* indicate directions of heme absorbance change at different wavelengths during the progression of the titrations. Structures of these molecules and other CYP126A1 ligands identified in this study are shown in Fig. 4. *B*, a UV-visible absorbance titration of CYP126A1 (4.6 μm) with compound **1** (*N*-isopropyl-*N*-((3-(4-methoxyphenyl)-1,2,4-oxadiazol-5-yl)methyl)-2-(4-nitrophenyl)acetamide). Compound **1** induces a P450 substrate-like type I blue shift of the heme Soret band from 418.5 nm (ligand-free) to 396 nm (nearly saturated with compound **1**). Isosbestic points in the titration are located at ∼405, 462, and 524 nm. The *inset* shows a plot of the maximal compound **1**-induced heme absorption change (Δ*A*_388_ − Δ*A*_419_) *versus* ligand concentration, with data fitted using a hyperbolic (Michaelis-Menten) function to give a *K_d_* of 18.3 ± 1.3 μm.

The UV-visible spectrum of oxidized (Fe^III^) CYP126A1 has major heme absorbance bands at ∼418.5 nm (Soret), 568.5 nm (α), and 539 nm (β). Reduction of CYP126A1 with sodium dithionite results in a Soret band shift to ∼412 nm with a Q-band feature at ∼544 nm. Binding of carbon monoxide to reduced CYP126A1 results in a Fe^II^-CO complex with a Soret peak at 448.5 nm and a Q-band feature at 549 nm (Fig. 2). The spectral features of the ferrous and Fe^II^-CO forms of CYP126A1 are consistent with the retention of cysteine thiolate as the proximal ligand to the P450 heme iron. However, the CYP126A1 Fe^II^-CO complex converts slowly to the cysteine thiol-coordinated P420 state (with Soret maximum at ∼423 nm) after ∼15 min, indicating a propensity for protonation of the cysteine thiolate in this state, as is also seen in other P450s, particularly in the absence of substrate (8, 18) (Fig. 2). Consistent with other *M. tuberculosis* P450s characterized to date, CYP126A1 binds a range of antifungal compounds containing imidazole heterocycles (7–10, 13, 14, 31) (Table 1). These compounds all produced type II (red) shifts in the Soret wavelength maximum (λ_max_) of the CYP126A1. For example, ketoconazole induces a Soret shift from 418.5 to 423.5 nm at apparent saturation, in addition to inducing smaller absorbance changes in the heme α- and β-band peaks (minor shifts to ∼569.5 and 540.5 nm, respectively, with notably decreased intensity of the α-band) (Fig. 3*A*). These spectral changes are consistent with ligation of the heme iron by a heterocyclic nitrogen atom. The CYP126A1 *K_d_* for ketoconazole is 0.34 ± 0.07 μm, whereas imidazole binds substantially more weakly (*K_d_* = 2.59 ± 0.06 mm). A common feature in the spectral titrations of CYP126A1 with imidazole group-containing azole drugs (ketoconazole, miconazole, econazole, clotrimazole, and imidazole itself) is the decrease in intensity of the red-shifted Soret band by ∼15–20% as the titration progresses to completion (Fig. 3*A*). The *K_d_* values for the binding of these compounds to CYP126A1 are given in Table 1 and compared with previously reported values for their binding to other *M. tuberculosis* P450s. None of the tested azole drugs containing triazole groups (fluconazole, voriconazole, and itraconazole) gave any convincing CYP126A1 Soret shifts, suggesting negligible or very weak binding to this P450.

**TABLE 1:** **Binding constants for azole drugs with CYP126A1 and other *M. tuberculosis* cytochrome P450 enzymes** The dissociation constants (*K_d_* values) for the binding of imidazole and several azole drugs to CYP126A1 and to various other *M. tuberculosis* cytochrome P450 enzymes were determined by UV-visible absorbance titration. Data for ligand binding are taken from the following references: CYP126A1 (this work), CYP144A1 (31, 72), CYP125A (7), CYP142A1 (8), CYP121A1 (19, 20), CYP130A1 (63), and CYP51B1 (14, 73). NB, no significant P450 spectral perturbation was observed upon the addition of the relevant azole drug. ND, there are no published data for the interaction of the relevant azole drug with the indicated P450 enzyme.

Azole inhibitor	*K_d_* for P450-azole drug binding						
	CYP126A1	CYP144A1	CYP125A1	CYP142A1	CYP121A1	CYP130A1	CYP51B1
	μ*m*						
Econazole	4.0 ± 0.6	0.78 ± 0.29	11.7 ± 0.7	4.6 ± 0.2	0.024 ± 0.006	1.93 ± 0.03	0.31 ± 0.04
Clotrimazole	3.9 ± 0.4	0.37 ± 0.08	5.3 ± 0.6	3.8 ± 0.9	0.073 ± 0.008	13.3 ± 0.6	0.18 ± 0.02
Miconazole	1.3 ± 0.2	0.98 ± 0.22	4.6 ± 0.4	4.0 ± 0.5	0.136 ± 0.021	1.70 ± 0.21	0.20 ± 0.04
Ketoconazole	0.34 ± 0.07	134 ± 5	27.1 ± 0.9	21 ± 4	3.41 ± 0.31	48.0 ± 1.5	3.57 ± 0.25
Fluconazole	NB	>10,000	43.2 ± 0.8	860 ± 108	8.61 ± 0.21	ND	5.82 ± 0.12
Voriconazole	NB	6510 ± 450	NB	NB	16.3 ± 2.1	ND	2.10 ± 0.16
4-Phenylimidazole	NB	280 ± 18	216 ± 5	12.0 ± 1.5	32.3 ± 2.2	ND	452 ± 27
Imidazole	2590 ± 60	2965 ± 275	536 ± 7	ND	ND	ND	11700 ± 900

##### Identification of Novel CYP126A1 Ligands and Their Binding Properties

**FIGURE 4.**
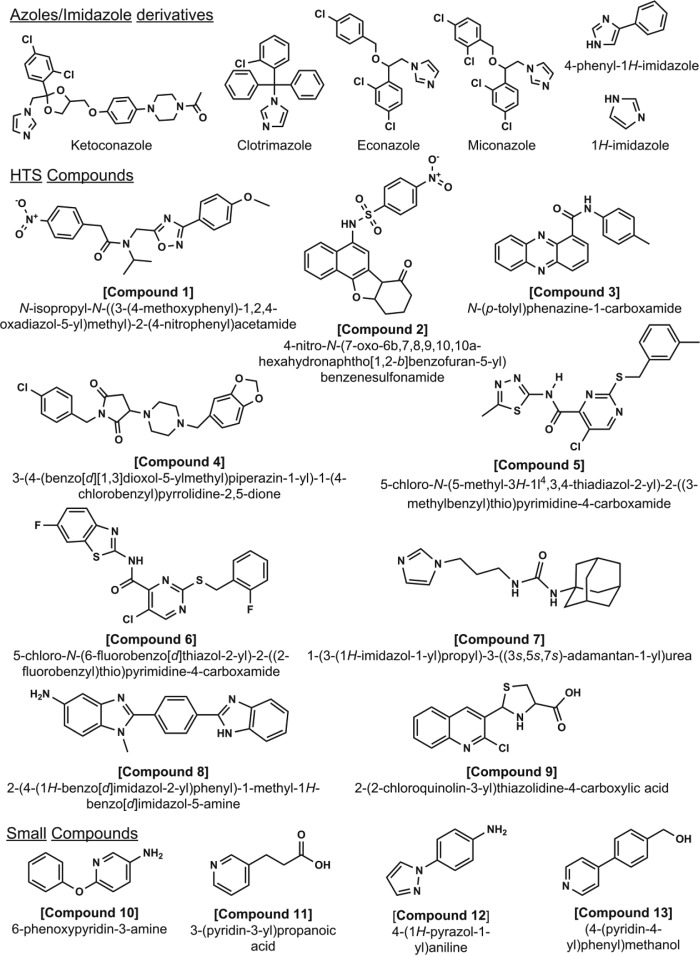
**Inhibitors and substrate-like ligands for CYP126A1.** Several ligands and potential substrates for CYP126A1 were identified using HTS methods. The figure shows (i) imidazole and imidazole-based azole drug inhibitors that coordinate the CYP126A1 heme iron, (ii) compounds identified by HTS that induce type I (compounds **1–6**, inducing HS heme iron accumulation) or type II (compounds **7–9**, coordinating the P450 heme iron) spectral shifts, and (iii) small nitrogen-containing molecules (compounds **10–13**).

To identify potential substrates and novel inhibitors of CYP126A1, a high throughput compound screen was undertaken. A series of ligands, compounds **1–9** (Fig. 4), were selected using UV-visible spectroscopy on the basis of their ability to induce either a substrate-like type I Soret shift (a blue shift, indicative of conversion of the low spin (LS) CYP126A1 toward HS) or a type II Soret shift (a red shift, usually indicative of heme iron coordination by a nitrogen or other atom from the compound). Compounds **1–6** each produced type I CYP126A1 P450 spectra, with the Soret band shifting from 418.5 to ∼395 nm. This is accompanied by a decrease in absorbance in the α/β-band region from ∼530–585 nm, and by increased absorbance at ∼650 nm characteristic for a cysteine thiolate-to-HS ferric heme iron charge transfer (CT) complex. Fig. 3*B* shows a spectral titration of CYP126A1 with compound **1**, with data fitted to give a *K_d_* of 5.62 ± 0.67 μm. Compounds **1–6** contain 3–5 rings, and compounds **1** and **2** both have a nitro-aromatic group at the end of the molecules. Affinity for compounds **1–6** varies in the range from ∼3 to 300 μm (Table 2). Compounds **7–9** induce type II spectra, and all contain groups that can ligate ferric heme iron through nitrogen atoms (*e.g.* imidazole, aromatic amine, and quinolone groups), with CYP126A1 *K_d_* values from ∼4 to 16 μm (Table 2). These compounds all induce heme spectral shifts similar to those seen with the imidazole antifungal drugs (Fig. 4).

**TABLE 2:** **Binding constants for compounds identified to bind CYP126A1 through high throughput screening studies** The dissociation constants (*K_d_* values) were determined by UV-visible absorbance titration. HTS compounds **1–6** induce type I (accumulation of HS heme iron) spectral shifts, whereas HTS compounds **7–9** reinforce the LS state of CYP126A1 through distal coordination of the heme iron via a nitrogen ligand in each case.

Type I	*K_d_*	Type II	*K_d_*
	μ*m*		μ*m*
Compound 1	18.3 ± 1.3	Compound 7	4.13 ± 0.61
Compound 2	2.85 ± 0.15	Compound 8	11.0 ± 0.6
Compound 3	56.1 ± 2.2	Compound 9	16.4 ± 0.7
Compound 4	65.2 ± 6.6		
Compound 5	6.15 ± 0.31		
Compound 6	25.3 ± 0.5		

##### EPR Spectroscopy of CYP126A1 and Ligand-bound Forms

X-band EPR spectroscopy was used to analyze the heme iron coordination environment of ferric CYP126A1 in its ligand-free state and when in complex with (i) HTS type I compounds **1–6** and (ii) HTS type II compounds **7–9**, as well as with a group of small compounds (compounds **10–13**) that were identified to bind with modest affinity (∼150–550 μm *K_d_*) to the CYP126A1 heme iron through amine, pyridine, or pyrazole nitrogens. Ligand-free CYP126A1 has a rhombic LS spectrum with a single major species, with g-values at 2.41 (g*_z_*), 2.24 (g*_y_*), and 1.92 (g*_x_*), consistent with one dominant coordination state from cysteine thiolate (proximal) and water (distal) ligands. These g-values are similar to those reported for other *M. tuberculosis* P450s, such as CYP125A1 (2.40/2.25/1.94), CYP142A1 (2.40/2.23/1.92), and CYP51B1 (2.44/2.25/1.91) (7, 8, 14).

**FIGURE 5.**
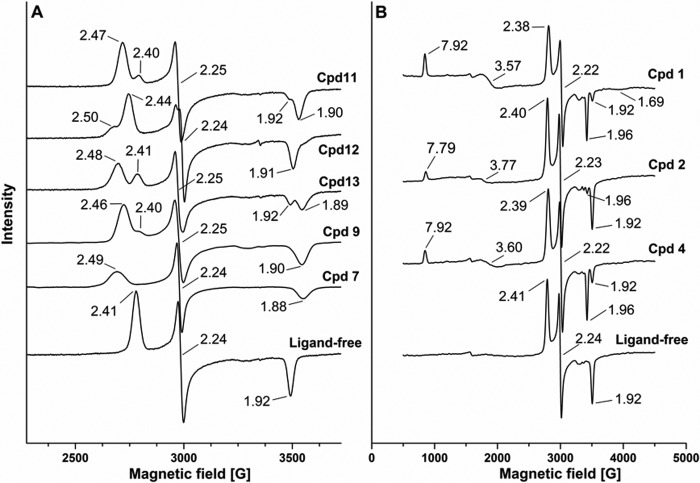
**EPR spectra of CYP126A1.** *A*, EPR spectra for CYP126A1 in its ligand-free form and for complexes with HTS compounds (compounds **7** and **9**) and other small molecules that ligate CYP126A1 heme iron via nitrogen ligands (compounds **11–13**). *B*, EPR spectra for HTS hits (compounds **1**, **2**, and **4**) that induce HS heme iron accumulation in CYP126A1. The g-values for the LS and HS forms of CYP126A1 are indicated in each case.

EPR spectra for CYP126A1 in complex with compounds **1–13** revealed substantial perturbations in several cases that were consistent with changes in distal heme iron coordination. HTS type II compounds **7–9** produced new LS EPR spectra consistent with distal ligation of CYP126A1 heme iron by a nitrogen atom. Similarly, the binding of HTS type I molecules resulted in new spectral signals indicative of the development of HS ferric heme iron as well as inducing some perturbations to the LS ferric signals. Selected EPR spectra are shown in Fig. 5.

CYP126A1 heme coordination by the four small nitrogen-containing molecules gave new LS spectra with g-values at 2.44/2.24./1.92 for 6-phenoxypyridin-3-amine (compound **10**); 2.47/2.25/1.90 for 3-(pyridin-3-yl)propanoic acid (compound **11**); 2.50/2.24/1.91 (minor) and 2.44/2.24/1.91 (major) for 4-(1*H*-pyrazol-1-yl)aniline (compound **12**); and 2.48/2.25/1.89 for (4-(pyridin-4-yl)phenyl)methanol (compound **13**). These EPR data suggest that direct coordination of heme iron may occur mainly through primary amine nitrogens in compound **10** and in the minor species in compound **12** (both with g*_z_* = 2.44). For the major species in compound **12** (g*_z_* = 2.50), pyrazole nitrogen may be the ferric iron ligand. The similar EPR spectral features for the CYP126A1 complexes with compounds **11** and **13** (g*_z_* = 2.47 and 2.48, respectively) suggest that pyridine nitrogen ligates heme iron in both of these complexes (Fig. 5*A*).

The type II hits from HTS studies also perturbed the LS ferric CYP126A1 EPR spectra, producing new species with g-values at 2.49/2.24/1.88 (compound **7**); 2.44/2.24/1.90 (compound **8**); and 2.46/2.25/1.90 (compound **9**) (Fig. 5*A*). The magnitude of change in the g-values for compounds **7–9** suggests heme coordination through the imidazole, aniline, and pyridine nitrogen atoms, respectively. For the type I HTS compounds, the development of HS ferric heme iron spectra was observed for compound **1** (g*_z_* = 7.92, g*_y_* = 3.57, g*_x_* = 1.69) and to a smaller extent for compounds **2** (7.79/3.77), **3** (7.85/3.63), and **4** (7.92/3.60). For compounds **2–4**, the HS g*_x_* signal was too small to be accurately assigned. No significant EPR spectral changes were seen for complexes with compounds **5** and **6**. EPR spectra for CYP126A1 complexes with compounds **1**, **2**, and **4** are shown in Fig. 5*B*. The relatively small accumulation of a HS EPR signal (compared with data collected by UV-visible titrations at ambient temperature) for many of the type I HTS compounds is consistent with data for several other P450s on binding substrate-like compounds and is probably due to the requirement for collection of heme EPR spectral data at 10 K (32). A P450 in which a much larger HS EPR signal is seen in the substrate-bound form is the alkene producing fatty acid decarboxylase OleT from a *Jeotgalicoccus* sp. OleT is converted to ∼95% ferric HS on binding arachidic acid (C20:0) and retains a large HS signal in the EPR spectrum, probably due to the presence of a rigid binding site for the fatty acid carboxylate close to the heme iron, leading to effective displacement of the water distal ligand from the OleT heme iron (32). For the HTS compounds that did induce formation of a HS ferric heme EPR signal, there was also an effect on the LS spectrum. As seen in Fig. 5*B*, there are shifts in the LS g-values to 2.38/2.22/1.96 (major), 1.92 (minor) for compound **1**; 2.40/2.23/1.92 (major), 1.96 (minor) for compound **2**; and 2.39/2.22/1.96 (major), 1.92 (minor) for compound **4**.

##### Magnetic Circular Dichroism (MCD) Spectroscopy of CYP126A1

**FIGURE 6.**
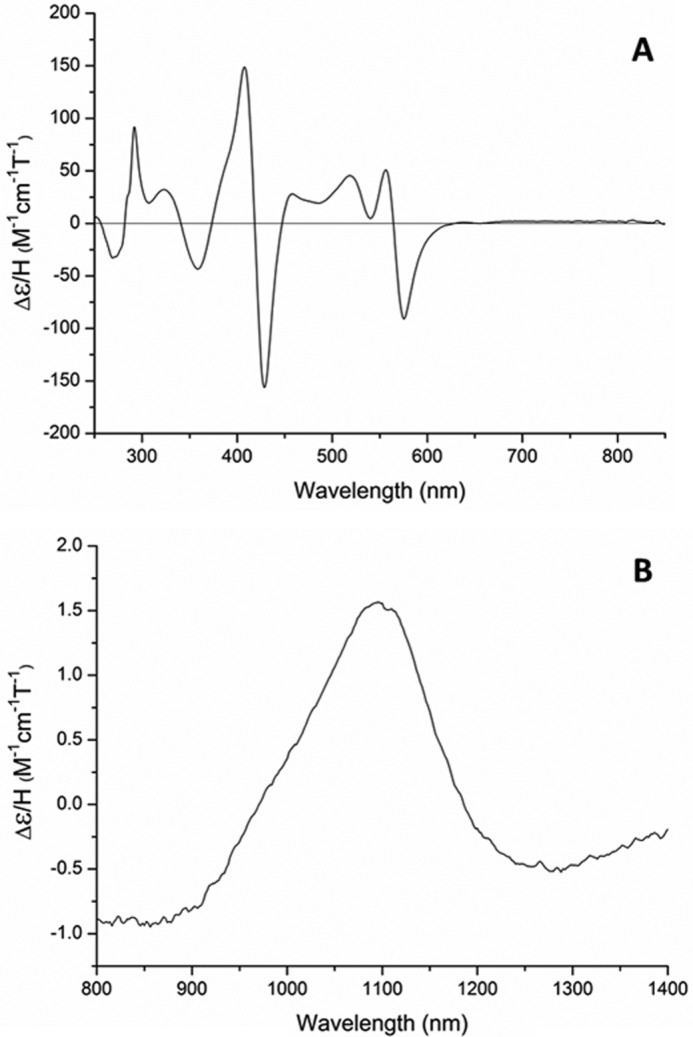
**MCD analysis of CYP126A1.** The MCD spectra for CYP126A1 are shown in the UV-visible region (*A*) and in the near-IR region (*B*). Spectra were collected at room temperature using a CYP126A1 concentration of 200 μm. Spectral features are consistent with a predominantly LS ferric heme iron with cysteine thiolate coordination.

MCD spectroscopy was used as a complementary method to EPR to probe electronic structure of CYP126A1. MCD data were collected in both the UV-visible (250–800 nm) and near-IR (800–1400 nm) regions. MCD features at different wavelengths in the UV-visible and near-IR regions can be used to probe heme structure and heme iron coordination (33, 34). UV-visible MCD spectra can be diagnostic for the spin and oxidation states of metal ions, and in heme proteins, the positions of the porphyrin macrocycle absorption bands are influenced by the heme iron. Porphyrin π-to-π* transitions give rise to MCD absorption features in the ∼300–600 nm region, and the mixing of the porphyrin-π and iron-d electronic levels enables UV-visible MCD to provide data on heme iron oxidation and spin state (35). Fig. 6*A* shows the UV-visible MCD spectrum of CYP126A1. The sharp feature at 292 nm is due primarily to contributions from tryptophan residues. Other major features in the heme spectrum (values in units of mm^−1^ cm^−1^) are located at 359 nm (−43.4) and 407.5 nm (+148.8), with a crossover at 418.5 nm in the Soret region, and at 520 nm (+48.7), 556.5 nm (+56.5), 546.5 nm (0), and 575.5 nm (−83.8) in the heme Q-band region. These features in the CYP126A1 UV-visible MCD spectrum resemble those of proteins with LS ferric hemes. For example, the UV-visible MCD spectrum of CYP126A1 is highly similar to that reported for the *M. tuberculosis* cyclodipeptide oxidase CYP121A1. A minor trough feature at ∼655 nm in the CYP126A1 UV-visible MCD spectrum indicates a very small proportion of HS ferric cysteine thiolate-coordinated heme iron (36).

A CYP126A1 MCD spectrum was also collected in the near-IR region, to report on the position of a ligand-to-metal CT transition that can be characteristic for the heme iron ligation state in the ferric form (Fig. 6*B*) (34). The CYP126A1 CT-band is located at ∼1097 nm. This position is consistent with those seen in other P450 enzymes (*e.g.* the fatty acid/fatty acyl-ACP oxidizing P450 BioI (CYP107H1) at 1090 nm and CYP121A1 at 1125 nm) (36, 37).

##### Determination of the Heme Iron Reduction Potentials of Substrate-free and Ligand-bound Forms of CYP126A1

The substrate-free form of CYP126A1 is predominantly LS in its resting ferric state. However, the binding of the type I HTS compounds **1–6** results in shifts toward HS heme iron, consistent with substrate-like properties. In many bacterial P450s, the substrate-induced HS shift is associated with a substantial increase in the P450 heme iron Fe^III^/Fe^II^ reduction potential (*e.g.* from −368 to −239 mV (*versus* normal hydrogen electrode (NHE)) for the *Bacillus megaterium* CYP102A1 (P450 BM3) heme domain on binding arachidonic acid and from approximately −300 to −170 mV for the *Pseudomonas putida* P450cam on binding d-camphor) (38, 39).

**FIGURE 7.**
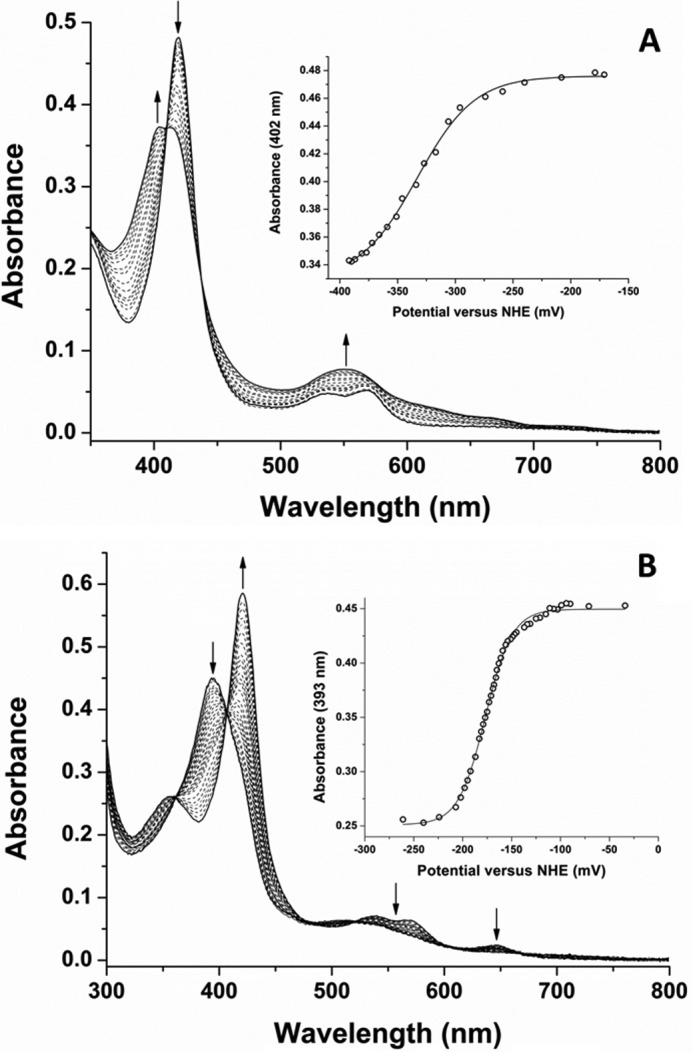
**Determination of the heme reduction potentials of ligand-free and compound 1-bound CYP126A1.** *A*, the *main panel* shows UV-visible absorbance spectral data from a spectroelectrochemical redox titration of ligand-free CYP126A1 in the potential range from ∼−170 to −395 mV (*versus* the NHE). The UV-visible Soret spectral maximum shifts from 418.5 nm toward ∼409 nm as the reductive titration approaches completion. In the Q-band region, the α- and β-bands appear at ∼568 and 539 nm in the oxidized enzyme, with the reduced CYP126A1 exhibiting a single absorption band at 550 nm. The absorbance *versus* potential data at 402 nm were fitted using the Nernst equation to give a midpoint potential for the heme iron Fe^III^/Fe^II^ couple of −332 ± 4 mV *versus* NHE (*inset*). *B*, a similar data set for the compound **1**-bound form of CYP126A1. In this case, the *main panel* shows P450 spectral data collected in the potential range from ∼−30 to −265 mV *versus* NHE. In the ferric state, the compound **1**-bound CYP126A1 has a Soret maximum at 394 nm, with Q-band maxima at 538 and 568 nm, and with a low intensity CT-band at ∼647 nm. The reduced form has a Soret spectral maximum at 421 nm, with loss of absorbance intensity in the Q-band region and for the CT species. Absorbance *versus* potential data were plotted at 393 nm and fitted using the Nernst equation to give a heme iron midpoint potential of −177 ± 3 mV *versus* NHE.

To analyze the influence of type I compound binding to CYP126A1, the heme iron redox potentials were determined for both the substrate-free CYP126A1 and the CYP126A1-compound **1** complex. The spectroelectrochemical redox titration for substrate-free CYP126A1 displays a Soret band transition from the oxidized form at 418.5 nm to a broad feature of lower intensity, centered at ∼409 nm, as the heme iron is reduced by progressive additions of sodium dithionite (Fig. 7*A*). There are clear isosbestic points in the titration (at ∼410 and 437 nm), indicating a simple conversion between the oxidized and reduced states without accumulation of an intermediate species. By comparison with other P450s, the blue shift of the Soret band and the spectral fusion of the α- and β-bands into a single Q-band feature at 550 nm is consistent with the retention of cysteine thiolate coordination in the ferrous heme iron form (40, 41). A plot of absorbance at 402 nm *versus* applied potential was fitted using the Nernst equation to give a midpoint potential for the CYP126A1 heme Fe^III^/Fe^II^ transition of −332 ± 4 mV *versus* NHE (Fig. 7*A*, *inset*).

In the case of the CYP126A1-compound **1** complex, the P450 is extensively HS in the ferric state, with a Soret maximum at 394 nm and a cysteine thiolate-to-ferric heme CT signal at 647 nm. The reduction of the heme iron occurs with a Soret band shift to 421 nm and with loss of the CT and spectral bleaching in the Q-band region. Isosbestic points in the redox titration at 406.5, 479, and 521 nm are again consistent with a simple two-state transition between oxidized and reduced forms of the CYP126A1-compound **1** complex (Fig. 7*B*). A plot of absorbance at 393 nm *versus* applied potential was again fitted using the Nernst equation, giving a heme Fe^III^/Fe^II^ midpoint potential of −177 ± 3 mV *versus* NHE (Fig. 7*B*, *inset*). Thus, binding of compound **1** results in a 155-mV increase in the CYP126A1 midpoint potential, consistent with heme iron reduction being favored in a substrate-bound form of the P450 (42).

##### Analysis of CYP126A1-mediated Oxidation of HTS Molecules

**FIGURE 8.**
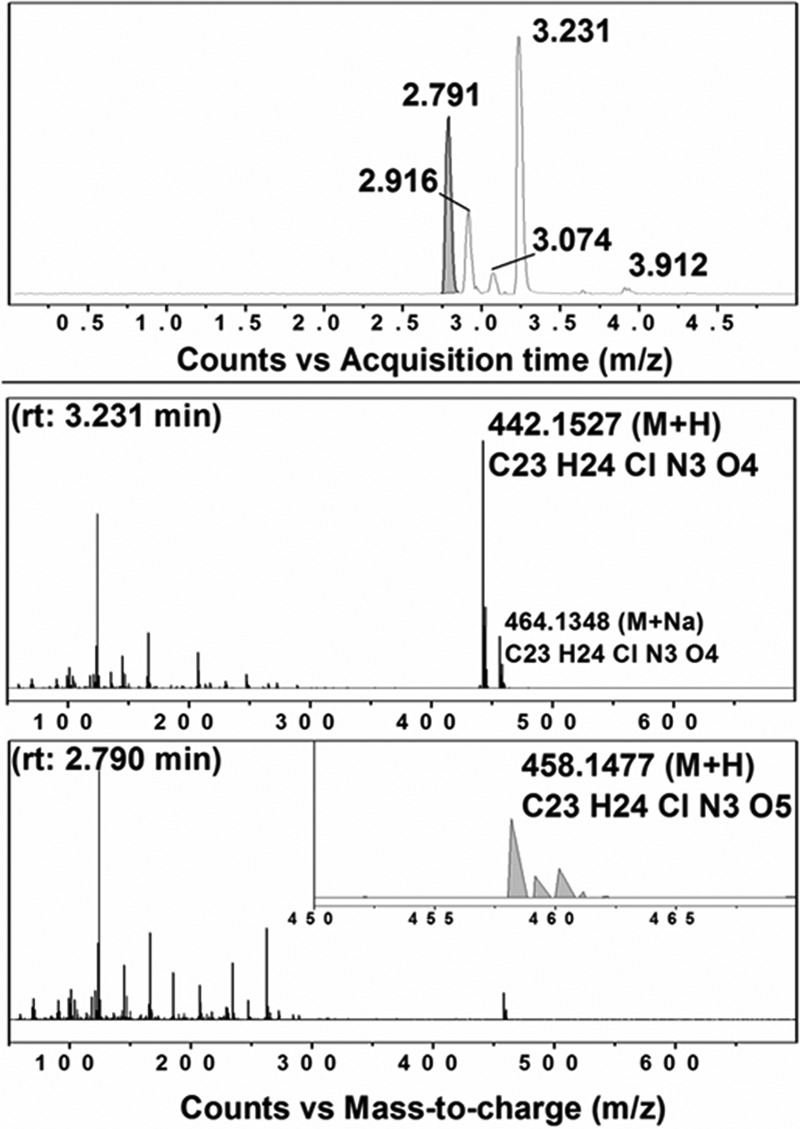
**CYP126A1-dependent oxidation of compound 4.** *A*, LC extracted ion chromatogram of CYP126A1 extracts following enzymatic incubations with compound **4**. Major peaks with retention times (*rt*) at 2.791 and 3.231 min are observed, with minor species at 2.916, 3.074, and 3.912 min. The peak at 2.791 min (*shaded* in *gray*) corresponds to the (+16) hydroxylation of compound **4**. *B*, mass spectrum following LC separation of the extracted major product at 3.231 min, with *m/z* 442.1528 (M + H) diagnostic of the parent substrate ion of compound **4** ((C_23_H_24_ClN_3_O_4_) + H)^+^ and a smaller species with *m*/*z* 464.1348 for the compound **4** sodium ion adduct. *C*, mass spectrum of the extracted 2.791-min peak showing the (+16) hydroxylation of compound **4** with *m*/*z* 458.1477 ((C_23_H_24_ClN_3_O_5_) + H). *Inset*, amplification of the peak *m*/*z* 458.1477, showing the ionization pattern for the hydroxylated form of compound **4**.

Studies to identify products of oxidation from several of the type I HTS compounds were done using two bacterial type NADPH-dependent redox systems comprising (i) *E. coli* flavodoxin reductase and (ii) *E. coli* flavodoxin or spinach ferredoxin. No turnover was detected with compounds **1** and **2**, both of which contain nitroaromatic groups. As discussed below, it is likely that these compounds act as inhibitors of CYP126A1 while simultaneously inducing a type I CYP126A1 heme spectral shift that is characteristic of substrate binding in most other P450s. Although evidence for oxygen incorporation (+16 addition to the parent species molecular mass) into a minor proportion of compounds **3** and **6** could be obtained from the small amounts of products resolved using LC-MS, there were insufficient products to identify the positions of oxidation. Extensive turnover of compound **5** did not produce any significant amount of oxidized product. However, in the case of compound **4** (3-(4-(benzo[*d*][1,3]dioxol-5-ylmethyl)piperazin-1-yl)-1-(4-chlorobenzyl)pyrrolidine-2,5-dione), a greater amount of oxidized product was formed. Although the precise position of oxidation could not be resolved fully by molecular fragmentation, it was clear that oxygen addition (hydroxylation) occurs on the 1,3-benzodioxole portion of the molecule (Fig. 8).

##### Analysis of CYP126A1 Oligomerization State and Ligand Binding Stoichiometry by Nano-ESI-MS

In previous studies, we used nano-ESI-MS to probe the oligomerization state of the *M. tuberculosis* P450 CYP121A1 and the enzyme's binding interactions with azole drugs. These studies revealed that ligand-free CYP121A1 is predominantly dimeric and that tightly binding azoles cause dissociation of the P450 dimer. In contrast, binding of the CYP121A1 substrate cYY gave a stable species that did not undergo gas phase dissociation (43).

**FIGURE 9.**
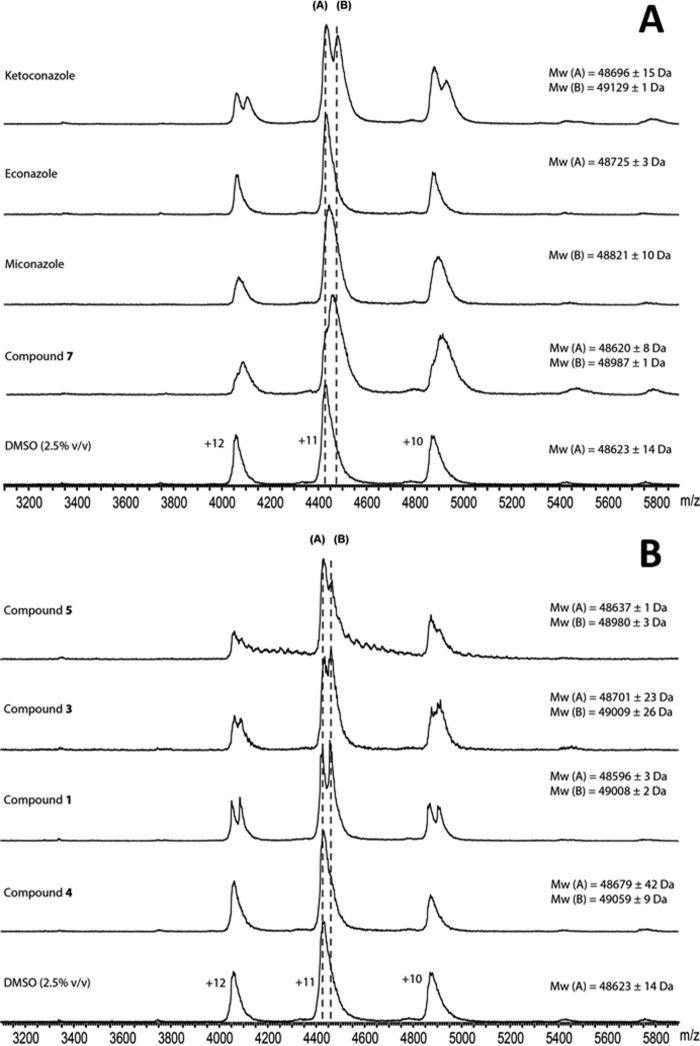
**Nano-ESI native mass spectra of CYP126A1-ligand complexes.** *A*, spectra from *bottom* to *top*: ligand-free CYP126A1 (10 μm) and CYP126A1 (10 μm) bound to compound **7**, miconazole, econazole, and ketoconazole (each 10 μm). *B*, from *bottom* to *top*: ligand-free CYP126A1 (10 μm); CYP126A1 bound to compounds **4**, **1**, and **3** (each at 10 μm); and CYP126A1 bound to compound **5** (50 μm). All samples contained 2.5% (v/v) DMSO-*d*_6_. *Dashed lines A* and *B* mark the *m*/*z* corresponding to the ligand-free enzyme and to CYP126A1 bound to one molecule of ligand, respectively. *, unbound and miconazole-bound CYP126A1 were unresolved at a 1:1 L/P ratio. The mass of the detected species (*B*) corresponds to 50% ligand-bound CYP126A1.

To assess the effect of ligand binding on CYP126A1 oligomerization state and to determine the stoichiometry of CYP126A1-ligand binding interactions, a series of nano-ESI native mass spectra were collected for CYP126A1 bound to the azole compounds ketoconazole, econazole, and miconazole and the HTS compounds **1**, **3**, **4**, **5**, and **7**. The effect of organic solvent on the ionization of CYP126A1 was first assessed. Low concentrations of DMSO-*d*_6_ (2.5%, v/v) caused an expected decrease in the charge state of the protein, shifting peaks for the CYP126A1 monomer from *m*/*z* 3200–4200 to *m*/*z* 4000–5100, but did not otherwise affect the quality of the spectra or the oligomerization state of the protein (Fig. 9) (44, 45). Ligand-bound spectra were collected at a minimum of three different ligand/protein ratios (from 0.5:1 to 25:1), depending on the *K_d_* values of the respective ligand. The stoichiometry of ligand binding was determined from the difference in the masses of peaks corresponding to ligand-bound CYP126A1 and ligand-free CYP126A1, divided by the molecular weight of the ligands. Ketoconazole and HTS compounds **1**, **3**, and **7** all exhibited clear binding at substoichiometric ligand/protein ratios (0.5:1) (Table 3). In contrast, econazole, miconazole, and compound **5** did not achieve complete occupancy of the CYP126A1 monomer until superstoichiometric ligand/protein ratios (5:1). These differences in the relative concentration of bound and unbound protein species observed in nano-ESI spectra are influenced by the binding affinity of the ligand, exposure of the binding site, and the type of intermolecular forces contributing to binding interactions, among other factors (46–48). Dissociation constants determined using ESI mass spectrometry have previously been reported to correlate reasonably well with *K_d_* values determined using other biophysical techniques for series of structurally similar ligands (46, 49–51). A greater proportion of ligand-bound CYP126A1 was observed at stoichiometric (1:1) ligand/protein ratios for ketoconazole and compounds **1**, **3**, and **7** compared with the other HTS and azole compounds. Because the solution phase *K_d_* values of the CYP126A1 ligands studied are all comparable, the differences in their gas phase affinity most likely reflect the nature of their binding interactions to the protein. Ligands whose binding affinity is predominantly driven by hydrophobic interactions show weaker than expected interactions in the gas phase, compared with compounds with polar or ionic binding contributions (46). The formation of ligand-protein complexes at substoichiometric ligand/protein ratios for ketoconazole and compounds **1**, **3**, and **7** indicates strong gas phase binding. This is consistent with the metal binding and hydrogen bonding interactions of these ligands with CYP126A1 that are observed by X-ray crystallography. In contrast, the azole drugs miconazole and econazole had disproportionately weaker affinity in nano-ESI experiments compared with their solution phase *K_d_* values, which is consistent with a hydrophobic mode of binding. The majority of compounds showed a maximum 1:1 binding stoichiometry over the concentration range tested. However, two of the HTS molecules (compounds **1** and **3**) were calculated to bind twice per CYP126A1 monomer at 5:1 ligand/protein ratios. No change in the oligomerization state of CYP126A1 was observed with any of the ligands.

**TABLE 3:** **Binding stoichiometry and relative affinity of HTS compounds for CYP126A1 determined using nano-ESI native mass spectrometry** Shown is the approximate ligand/CYP126A1 protein (L/P) binding stoichiometry for selected HTS hits (compounds **1**, **3**, **4**, **5**, and **7**) and azole drugs (ketoconazole, miconazole, and econazole) at 0.5:1, 1:1, and 5:1 L/P ratios. The final column shows the percentage occupancy of the ligand at a 1:1 L/P molar ratio. Two molecules of compounds **1** and **3** can bind to CYP126A1 at a 5:1 L/P ratio. ND, no ligand-bound CYP126A1 complex was detected at the given L:P ratio, or spectra were only collected at higher L/P ratios.

Ligand	*M*_r_	Ligand binding stoichiometry			CYP126-ligand complex (1:1)
		L/P 0.5:1	L/P 1:1	L/P 5:1	
					%
Ketoconazole	531.434	0.3	1	1	46
Miconazole	416.123	ND	0.5*^a^*	1	ND*^a^*
Econazole	381.681	ND	0.3	1	0
Compound 1	410.43	1	1	2	51
Compound 3	313.36	1	1	2	52
Compound 4	441.912	ND	1	1	35
Compound 5	391.892	ND	ND	1	ND
Compound 7	302.422	0.2	1	1	63

*^a^* Unbound and miconazole-bound CYP126A1 were unresolved at a 1:1 L/P ratio. The mass of the detected species corresponds to 50% ligand occupancy.

##### Crystal Structure of CYP126A1 in the Imidazole-bound Form

**FIGURE 10.**
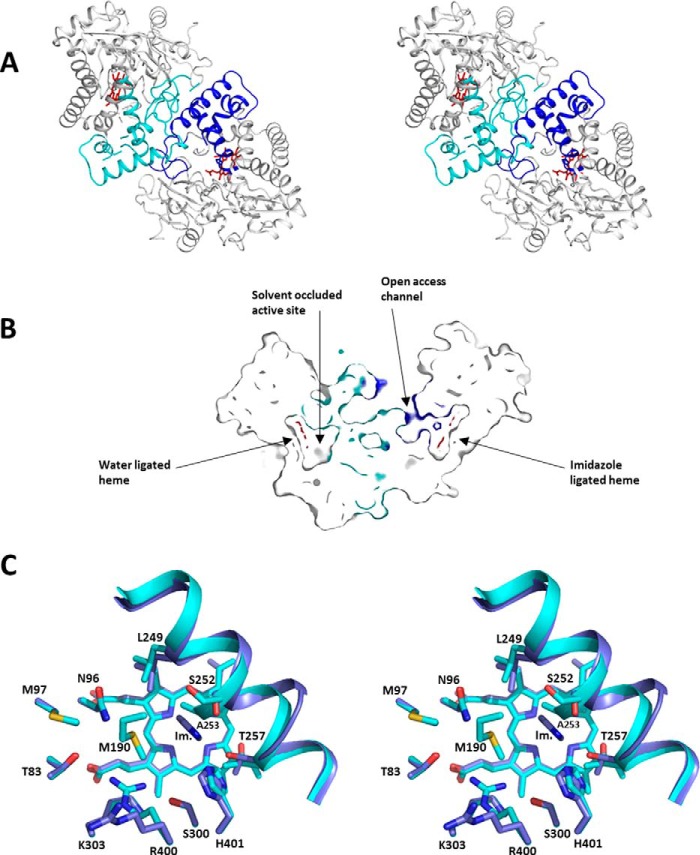
**The crystal structure of CYP126A1.** *A*, *stereoview* of the CYP126A1 dimer structure with the N-terminal region of the I-helix as well as the F- and G-helices and the BC-loop region, *colored* in *blue* for the imidazole-bound monomer and in *cyan* for the ligand-free monomer. *B*, a cross-section of the CYP126A1 dimer through both heme groups, revealing that the imidazole-bound monomer has an open active site with access to solvent, whereas the opposite monomer has a closed active site. *Color coding* is as in *A. C*, *stereoview* of an overlay of both active sites of the CYP126A1 dimer. Key residues are shown in atom *colored sticks*, with *blue carbons* for the imidazole-bound monomer and *cyan carbons* for the ligand-free monomer.

The structure of the ligand-free form of CYP126A1 (PDB code 5LIE) was determined by molecular replacement using the structure of the P450 EryF (CYP107A1), a 6-deoxyerythronlide B hydroxylase from the erythromycin biosynthesis pathway in *Saccharopolyspora erythraea* (PDB code 1OXA) (52). The CYP126A1 structure exhibits the characteristic P450 fold, and the ligand-free structure contains a dimer in the asymmetric unit (Fig. 10). The dimer interface is made by the BC- and FG-loop regions, both of which are associated with substrate entry into P450 enzymes. One monomer (chain A) forms a “closed” state with a relatively solvent-inaccessible active site and has water as the sixth heme ligand. The other monomer is bound to imidazole as the sixth heme ligand (probably retained after the nickel affinity purification step in a proportion of the P450 molecules). In contrast to the ligand-free active site, the imidazole-bound form (chain B) contains a clear solvent access channel (the “open” state). The access channel to the bound imidazole is opened due to disorder of the FG-loop region (residues 177–194). In comparison, the FG-loop region of chain A is ordered but explores, in part, the chain B active site region. Hence, it is possible that ligand binding to chain B affects the conformation of chain A. An overlay of both monomers reveals that imidazole binding is linked to a reorientation of the N-terminal region of the I-helix at residues 240–257, moving Ala-253 by 2.7 Å to avoid severe steric clashes with the heme sixth ligand. Because the F- and G-helices are involved in hydrophobic contacts with this particular region of the I-helix, this I-helix relocation leads to an accompanying shift in orientation of the FG region. Whereas the chain B FG-loop is disordered, the chain A FG-loop chain region makes contact with the chain B I-helix N-terminal region at residues 194–196.

The asymmetric behavior of the CYP126A1 dimer can be rationalized by the fact that a putative A-A symmetric dimer (where both chains adopt the chain A conformation) is not possible due to a severe steric clash of the respective FG-loop regions. In other words, the ordered FG-loop region in chain A at the dimer interface obscures the putative FG-loop docking region for chain B. Because imidazole binding leads to reorientation of the I-helix and consequently the FG-helix, it is possible that imidazole binding to one monomer leads to the active site closure of the second monomer. It is interesting to note that a putative B-B symmetric dimer (where both chains adopt the “open” chain B conformation) also appears possible.

Given the flexibility of the P450 fold, it is difficult to predict substrate identity from P450 structures. In the case of CYP126A1, the ligand binding pocket is not exclusively lined by hydrophobic residues (Met-190, Leu-249, and Ala-253) but also contains Thr-83, Asn-96, Ser-300, Lys-303, Arg-400, and His-401, suggesting that the physiological substrate probably exhibits hydrophilic properties. For chain B, the imidazole ligand is positioned between I-helix residues Leu-249 and Thr-257 and is stacked against Ala-253. In this case, the side chain of Arg-400 has reoriented and points away from the active site.

##### Crystal Structure of CYP126A1 in Complex with Inhibitor and Substrate-like Molecules

**FIGURE 11.**
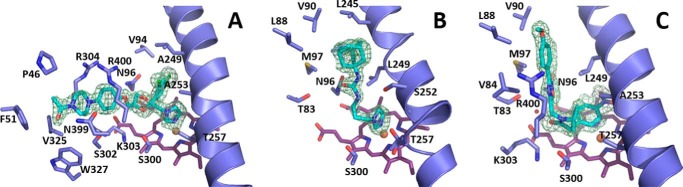
**Active site structures of CYP126A1-ligand complexes.** Detailed views of the active sites of CYP126A1 are shown from the structures of the complexes with the type II inhibitors ketoconazole (*A*) and compound **7** (*B*), and the type I substrate mimic compound **1** (*C*). Key residues are shown in atom *colored sticks*, with the m*F_o_* − D*F_c_* omit electron density for the various ligands contoured at 3 σ. The N-terminal region of the I-helix is shown in a schematic. Compounds **1** and **7** bind in a channel that follows the general direction of the I-helix, whereas ketoconazole extends away from the heme in an orthogonal direction.

To further establish the nature of CYP126A1 protein-ligand interactions and the effects these have on protein oligomerization/conformation, we determined the crystal structures of three distinct inhibitor complexes through co-crystallization. Fig. 11 shows the binding modes of ketoconazole (PDB code 5LI8), the smaller imidazole-containing compound **7** (PDB code 5LI7), and compound **1** (PDB code 5LI6) in the active site of CYP126A1. These images show that compounds **1** and **7** bind in a channel that follows the general direction of the P450 I-helix. However, ketoconazole binds in a different manner and extends from the heme in an orthogonal direction to the other two molecules. The distinctive binding mode for ketoconazole has ramifications for the structural organization of CYP126A1.

**FIGURE 12.**
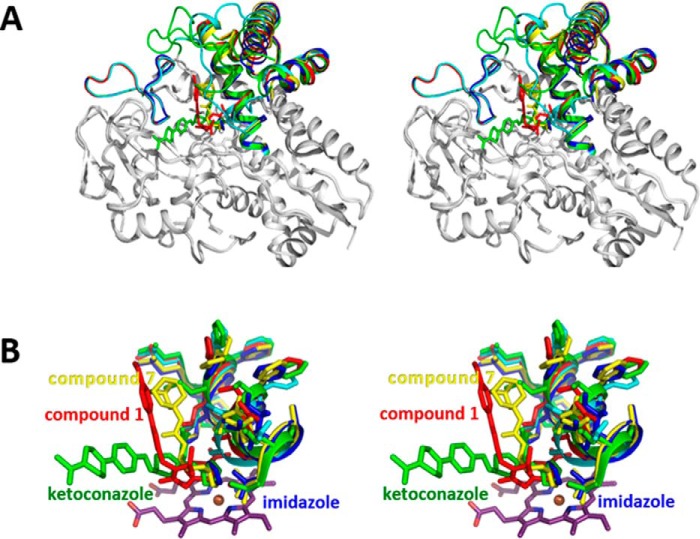
**Structures of CYP126A1-ligand complexes.** Overlays of the various CYP126A1-ligand complex structures are shown. *A*, *stereoview* of an overlay of the CYP126A1 ligand complex structures together with the ligand-free monomer structure (in *cyan*). The ligand complexes are *colored blue* for imidazole, *yellow* for compound **7**, *red* for compound **1**, and *green* for ketoconazole. Only those regions affected in conformation by ligand binding are in *color. B*, *stereoview* of an overlay of the various CYP126A1-ligand complex active sites. *Color coding* is as in *A*. The N-terminal region of the I-helix is shown, revealing that highly ligand-specific changes occur. These are transmitted through to the F- and G-helices that are in direct contact with this region.

The crystal structure of CYP126A1 in complex with the azole drug ketoconazole revealed that a large scale reorientation of the BC-loop region has occurred in response to ligand binding, disrupting the putative dimer interface observed in the imidazole-bound form (Fig. 12). Furthermore, the N-terminal region of the I-helix and the associated FG-helices display comparatively more modest reorientations as a consequence of ligand binding. The ketoconazole dichlorobenzene moiety occupies the region previously filled by Leu-249, whereas the extended tail of the molecule displaces the BC-loop residues Val-84 and Leu-85, resulting in their forming hydrophobic contacts with the FG-helices.

In contrast, the binding of the smaller imidazole-containing compound **7** does not lead to BC-loop reorientation, and a putative CYP126A1 dimer in complex with compound **7** in both of its active sites is observed in the asymmetric unit. The overall conformation is similar to the proposed B-B symmetric dimer, with both chains adopting a conformation resembling the imidazole-bound monomer. In addition to heme iron coordination by the compound **7** imidazole group, further contacts with CYP126A1 are limited to hydrogen bonds with Asn-96 and Ser-252 and with the ligand urea moiety, whereas the adamantyl group remains largely solvent-exposed (Fig. 12).

Unlike the previous ligands, compound **1** does not contain an azole ring and instead elicits a type I heme shift suggestive of substrate-like ligand binding. The crystal structure of CYP126A1 in complex with compound **1** reveals that the ligand is bound with the nitrobenzene moiety placed directly above and parallel to the heme porphyrin plane (Fig. 12). This effectively ensures that the heme iron sixth ligand water is displaced, explaining the observed spectroscopic effect with HS heme iron formation. As observed for the azole-containing ligands, the binding of compound **1** elicits a shift in the N-terminal region of the I-helix to accommodate the nitrobenzene moiety, accompanied by the reorientation of the FG-helix region. As observed for compound **7**, the BC-loop region appears unperturbed by compound **1** binding. However, the putative dimer interface observed in the CYP126A1-compound **1** complex is distinct in nature. Although still composed of interactions between the FG- and BC-loop regions, the respective orientation of both monomers is altered. Although compound **7** occupies a binding pocket similar in nature to that observed for compound **1**, it appears that small changes in the conformations of residues lining the compound **7** binding pocket (Gln-86 and Arg-400) lead to distinct packing interactions with the FG-helices of the opposite dimer. Crystallographic data are presented in Table 4.

**TABLE 4:** **Data reduction and final structural refinement statistics for the *M. tuberculosis* H37Rv CYP126A1 and drug complexes** Values in parentheses are for the highest resolution shell. NA, not applicable.

Parameters	CYP126A1	CYP126A1 ketoconazole	CYP126A1 compound 1	CYP126A1 compound 7
**Data collection**				
Space group	P2_1_2_1_2_1_	C2	P2_1_	P2_1_2_1_2_1_
Cell dimensions				
*a*, *b*, *c* (Å)	58.93, 70.05, 233.66,	118.23, 59.66, 69.84,	69.62, 59.66, 118.47,	59.25, 69.91, 226.14
α, β, γ (degrees)	90, 90, 120	90, 93.2, 90	90, 98.03, 90	90, 90, 90
*R*_merge_ (%)	0.034 (0.423)	0.048 (0.666)	0.031 (0.842)	0.024 (0.829)
*I*/σ*I*	13.4 (1.7)	19.1 (2.2)	11.9 (1.8)	20.1 (2.6)
Completeness (%)	99.6 (99.0)	92.47 (59.1)	99.5 (96.8)	99.8 (100)
Redundancy	3.7 (3.9)	3.9 (3.3)	4.0 (3.4)	7.3 (5.4)

**Refinement**				
Resolution (Å)	46.99–1.80 (1.86–1.80)	43.86–1.83 (1.88–1.83)	32.14–1.95 (1.99–1.95)	57.31–1.58 (1.62–1.58)
No. of reflections	90,448 (8427)	37,754 (1747)	66,993 (4759)	122,489 (8783)
*R*_work_/*R*_free_	16.62/20.30 (18.0/23.9)	15.7/19.6 (24.0/33.5)	18.88/24.2 (31.6/38.9)	0.169/20.34 (26.0/31.5)
No. of non-hydrogen atoms	7079	3594	6948	7064
Mean *B* factor (Å^2^)				
Protein	24.6	17.6	21.2	20.1
Non-heme ligand	21.7	17.1	18.7	23.4
Solvent	34.8	24.7	31.3	32.9
Root mean square deviations				
Bond lengths (Å)	0.016	0.025	0.022	0.027
Bond angles (degrees)	1.444	2.001	1.775	2.056
Ligand-iron coordination distance (Å)	2.25	2.0	NA	2.01
Dimer interface (Å^2^)	5830	NA	3990	4160
Ramachandran statistics (%)				
Preferred region	96.1	96.7	96.6	95.7
Allowed region	3.4	3.0	3.1	4.0
Outliers	0.5	0.3	0.3	0.3
**PDB code**	5LIE	5LI8	5LI6	5LI7

## Discussion

Orthologs of the *M. tuberculosis CYP126A1* gene are widely distributed among both pathogenic (*e.g. Mycobacterium ulcerans* Agy99, 80% amino acid sequence identity) and non-pathogenic (*e.g. M. smegmatis* mc^2^155, 79% identity) mycobacteria. This suggests an important conserved function in these organisms. Related P450s are also found in other actinobacteria (*e.g. Streptomyces* sp. Eco86, 43% identity) and also among the myxobacteria (*e.g. S. cellulosum* So ce56, 42% identity; and *Stigmatella aurantiaca* DW4/3-1, 41% identity), again suggesting that a common function may be retained in diverse bacteria (53). Phylogenetic studies indicate that CYP126A1 is most closely related to CYP125A1, CYP142A1, and CYP124A1 from *M. tuberculosis* H37Rv, all of which have been implicated in host cholesterol metabolism (6–9, 54, 55). However, we could find no evidence for cholesterol binding to CYP126A1 by UV-visible spectral titration. Although there are relatively few data available on *CYP126A1* from transcriptomics and microarray studies, the *CYP126A1* gene is not essential for the *in vitro* growth of the virulent *M. tuberculosis* H37Rv strain. However, its importance in the infective state remains uncertain (56). CYP126A1 protein is clearly produced in *M. tuberculosis* and was identified in *M. tuberculosis* H37Rv cell lysates by mass spectrometry. It was not identified in bacterial culture filtrate or membrane fractions, consistent with it being a soluble, cytosolic protein (57, 58). Moreover, a computational analysis of predicted *M. tuberculosis* target proteins and their druggability ranks CYP126A1 (and other *M. tuberculosis* P450s) in the top 50 potential targets among more than 1500 candidate *M. tuberculosis* proteins (59).

To characterize in more detail the structural and biochemical properties of CYP126A1, we purified the P450 using an *E. coli* expression system and demonstrated by mass spectrometry that the protein was intact. Nano-ESI native mass spectrometry indicated that the enzyme is predominantly monomeric but has a propensity to form higher dimeric, trimeric, and tetrameric species at relatively low concentrations (Fig. 1). This property probably underpins the formation of CYP126A1 dimer crystals (see below). UV-visible spectroscopy confirms typical heme spectral features in CYP126A1. A slow collapse of the P450 form of the CYP126A1 Fe^II^-CO complex at 448.5 nm to the P420 state at 423 nm occurs, and this phenomenon is also observed in other *M. tuberculosis* P450s (8, 13, 14). On the basis of previous studies, it is expected that the thiolate-coordinated P450 state would be stabilized by the binding of its natural substrate (8, 18). Supporting this conclusion, the stabilization of the thiolate-coordinated ferrous form of CYP126A1 is evident in the redox titration of the P450 bound to the type I screening hit compound **1**. The blue Soret shift seen on CYP126A1-compound **1** heme iron reduction, along with spectral “fusion” of the Q-bands, is characteristic of retention of thiolate coordination in the ferrous state, whereas a Soret red shift (*e.g.* to 423 nm in the case of *M. tuberculosis* CYP51B1) with shifted α- and β-band features is typical for cysteine thiol-coordinated ferrous heme iron (14, 38). The redox titration also reveals an ∼155-mV increase in the reduction potential of the CYP126A1 heme iron Fe^III^/Fe^II^ couple, an effect probably dominated by the extensive conversion of the CYP126A1 ferric heme iron to the HS state (38, 39). However, the significant structural rearrangements accompanying the binding of compound **1** (Fig. 11) also alter the CYP126A1 heme environment and are likely to influence the heme redox potential.

CYP126A1 binds avidly to a number of azole drugs but shows a clear preference for binding azole antifungal drugs with imidazole rather than triazole groups. Azole binding to CYP126A1 induces a red (type II) Soret band shift with a distinctive decrease of Soret absorbance intensity (by ∼15–20%) as the titrations progress to completion (Fig. 3). An interesting pattern of azole drug selectivity among *M. tuberculosis* P450s emerges from data presented in Table 1. First, it is clear that in the case of CYP126A1, CYP144A1, and CYP121A1, the affinity for the imidazole-containing azole drugs is markedly greater than those for the triazole-containing drugs tested. Indeed, no significant type II Soret shifts were observed for CYP126A1 with fluconazole, voriconazole, or itraconazole. The CYP144A1*K_d_* values for econazole, clotrimazole, and miconazole are submicromolar, whereas those for the triazoles fluconazole and voriconazole are >10 and 6.5 mm, respectively. CYP121A1 binds fluconazole and voriconazole more tightly (*K_d_* values are 8.61 and 16.3 μm, respectively), but affinity remains ∼120–680-fold weaker than for econazole and miconazole. However, there is much less discrimination between the imidazoles and triazoles in at least CYP51B1 and CYP125A1, although voriconazole does not bind the heme iron in CYP125A1. For CYP51B1, the binding of fluconazole and voriconazole is quite tight (*K_d_* = 5.82 and 2.10 μm, respectively) and only 12–32-fold weaker than that for clotrimazole, the tightest binding imidazole drug. Ketoconazole displays similar affinity (*K_d_* = 3.57 μm) to the triazole drugs in CYP51B1. It is known that that CYP51B1, CYP125A1, and CYP142A1 are all sterol-oxidizing P450s in *M. tuberculosis*, whereas no such role has been assigned to CYP126A1 or the other *M. tuberculosis* P450s in Table 1. The binding of fluconazole and voriconazole to CYP126A1 is not detectable, whereas at least fluconazole binds each of three known sterol-metabolizing *M. tuberculosis* P450s with good to moderate affinity. It can thus be speculated that, despite apparent phylogenetic relationships between CYP126A1 and sterol-metabolizing *M. tuberculosis* P450s, CYP126A1 has taken a distinct evolutionary path and that its role in pathogenic and non-pathogenic mycobacteria (and other bacteria) may be unrelated to metabolism of host or environmental sterols. With regard to the effectiveness of the various azole drugs in inhibiting mycobacterial growth, preceding studies (60) showed that the triazoles fluconazole and itraconazole had no significant effect on *M. tuberculosis* growth *in vitro*, whereas ketoconazole had an MIC of 8–16 μg/ml against the virulent *M. tuberculosis* H37Rv strain. Our own studies confirmed the greater potency of the imidazole-containing azole drugs, with fluconazole proving ineffective against *M. tuberculosis* H37Rv, whereas econazole (8 μg/ml), miconazole (8 μg/ml), clotrimazole (11 μg/ml), and ketoconazole (16 μg/ml) all showed good MIC values (20). Imidazole azole drug potency was even greater against the laboratory strain *M. smegmatis* mc^2^155 in most cases, with MIC values of <0.1 μg/ml for econazole, 0.1 μg/ml for clotrimazole, and 1.25 μg/ml for miconazole, compared with >100 μg/ml for the triazole fluconazole (19). The enhanced azole potency against *M. smegmatis* mc^2^155 may reflect greater drug permeability as a consequence of altered glycolipid composition in the cell envelope (61).

In work to identify novel type I and type II compounds binding to CYP126A1, we undertook a large (∼40,000) compound screen and identified several molecules that induce CYP126A1 heme Soret shifts consistent with substrate-like (HS heme iron accumulation with Soret blue shift) or heme iron-ligating (Soret red shift, similar to that seen for azole drug binding) properties. The compound hits selected for analysis were all extended molecules containing between 2 and 6 aromatic or non-aromatic ring structures (Fig. 4). Although these bulky compounds do not provide immediate clues to the identity of physiologically relevant substrates, type I CYP126A1 spectral shifts were obtained in several cases, and evidence of P450-mediated substrate oxidation of HTS type I compounds was obtained in three cases, with the clearest results obtained with compound **4**.

Crystal structures were determined for CYP126A1 in the absence of added ligand and for CYP126A1 in complex with ketoconazole and with HTS molecules compound **1** (type I hit) and compound **7** (type II hit). The imidazole group of compound **7** clearly ligates the heme iron, whereas the nitrobenzene moiety of compound **1** is placed directly above the heme plane and displaces the distal water, inducing adoption of the HS ferric state in CYP126A1. No evidence of oxidation of either compound **1** or compound **2** (which both possess a terminal 4-nitrophenyl group) could be obtained in turnover studies, and it thus appears that these molecules “moonlight” as substrates but are not readily oxidized and instead act as P450 inhibitors. A small number of nitroaromatic compounds were identified as inhibitors of the P450-like, cysteine thiolate-coordinated nitric-oxide synthase enzymes (62). However, there is no available evidence for their binding in a similar mode to that shown here structurally for compound **1**. The similar binding modes for both compounds **1** and **2** to CYP126A1 suggest that binding studies with other nitroaromatic compounds could identify more potent inhibitors of CYP126A1.

The CYP126A1 structures reveal a dynamic molecule, with the BC and FG regions clearly affected by ligand binding. These regions are also involved in the formation of CYP126A1 dimers observed in some crystal forms, as indicated by PISA analysis (see Table 4 for corresponding dimer interface size), establishing a putative link between ligand binding and protein oligomerization. However, it remains unclear whether the same types of interactions lead to CYP126A1 dimerization in the solution state. The malleable nature of CYP126A1 (as demonstrated by its ability to bind to a range of structurally diverse inhibitors and substrate-like molecules) suggests that the nature of the physiological substrate does not necessarily reflect the shape of the ligand-free CYP126A1 structure. However, the nature of the residues that line the various active site regions does suggest that the physiological substrate contains polar moieties that might establish interactions with residues such as Thr-83, Asn-96, Ser-300, Lys-303, Arg-400, and His-401. Ketoconazole binding reorients the BC-loop region of CYP126A1 and disrupts the dimer interface, resulting in a binding mode for ketoconazole that is approximately orthogonal to those for HTS compounds **1** and **7**, which extend upward from the heme, close to the path followed by the I-helix (Fig. 11). The binding modes of these molecules are compatible with CYP126A1 dimer formation in the crystal, although differences in the dimer interface regions are observed. The ability of CYP126A1 to form crystallographic dimers is reminiscent of properties reported for the *M. tuberculosis* CYP130A1 P450, with similar regions of the P450s forming the dimer interfaces. However, whereas CYP130A1 crystallizes as a monomer in the “open” conformation when ligand-free, the econazole-bound form has a “closed” conformation in a dimeric state (63). In contrast, the ketoconazole-bound CYP126A1 is a crystallographic monomer, whereas the ligand-free form of CYP126A1 is a dimer containing both “open” and “closed” monomers.

In conclusion, we present the first biochemical and structural studies of the *M. tuberculosis* P450 CYP126A1, revealing novel substrates and inhibitors for the enzyme, defining its relatively polar active site and its ability to adapt structurally to facilitate the binding of bulky ligands. Future work will be directed at the identification of the natural substrate(s) for this widely conserved P450 enzyme.

## Experimental Procedures

### 

#### 

##### Cloning and Production of a CYP126A1 Expression Plasmid Construct

The gene encoding CYP126A1 (*Rv0778*) from the *M. tuberculosis* H37Rv strain was amplified by PCR from a *M. tuberculosis* H37Rv chromosomal cosmid DNA library (supplied by Dr. Roland Brosch, Institut Pasteur, Paris). The *CYP126A1*-containing bacterial artificial chromosome clones were prepared using standard PCR amplification protocols with *Pfu Turbo* DNA polymerase (Agilent, Cheadle, UK) used to amplify the gene. The *M. tuberculosis* H37Rv genome sequence was used to design primers to generate the *Rv0778* gene for insertion into the pET15b plasmid vector using the forward primer 5′-GCGGCAGCCAT**ATG**ACTACCGCCG-3′ and the reverse primer 5′-GCGCGGAGGC**TA****G**GATCCGGCTGC-3′ (Merck-Millipore, Watford, UK). The underlined letters in the forward and reverse primers indicate engineered NdeI and BamHI restriction endonuclease sites, respectively. The boldface letters indicate the start (ATG) and stop (TAG) codons, respectively. The gene amplification conditions used were 95 °C for 2 min; 30 cycles of 95 °C for 45 s, 62 °C for 30 s, and 72 °C for 1.5 min; and then a final polymerization step of 72 °C for 10 min. The PCR product was then digested using NdeI/BamHI and cloned into pET15b using a Quick Ligation Kit (New England Biolabs, Hitchin, UK) to generate the pCYP126A1 construct.

##### Expression and Purification of CYP126A1

The production of CYP126A1 protein was achieved by transforming *E. coli* strain BL21 Gold (DE3) (Agilent) or C41 (DE3) (Merck-Millipore) with the pCYP126A1 plasmid construct. Gene expression used an isopropyl β-d-thiogalactopyranoside (IPTG)-inducible T7 RNA polymerase/promoter system. IPTG-dependent expression of the T7 RNA polymerase from a chromosomally integrated gene copy in C41 (DE3) resulted in T7 RNA polymerase-dependent transcription of the *CYP126A1* gene in pET15b. Production of CYP126A1 protein was typically done in ∼15-liter cultures of 2xYT growth medium (ForMedium, Hunstanton, UK). The culture medium was distributed among 24 2-liter conical flasks, each containing ∼600 ml of growth medium and ampicillin (50 μg/ml) for plasmid selection. The medium was inoculated with 6 ml of *E. coli* transformant cells from an overnight culture of cells grown in the same medium. The cells were grown at 37 °C with agitation (200 rpm) until an *A*_600_ of 0.5 was reached, and then the growth temperature was decreased to 23 °C, and bacterial cell growth continued until an *A*_600_ of 0.7 was reached. 100 μm IPTG was then added to induce *CYP126A1* gene expression, along with 100 μm Δ-aminolevulinic acid to promote heme synthesis and its incorporation into the P450. The transformant cells were then grown for a further 36 h under the same conditions. The cells were harvested by centrifugation at 6000 × *g* for 10 min at 4 °C using a JLA-8.100 rotor in an Avanti J-26 XP centrifuge, after which the supernatant was discarded and cell pellets were resuspended in ∼300 ml of ice-cold 50 mm potassium phosphate (KP_i_, pH 8.0) containing 250 mm NaCl and 10% glycerol (buffer A). The protease inhibitors PMSF (1 mm), benzamidine hydrochloride (1 mm), and six cOmplete^TM^ EDTA-free tablets (Roche Diagnostics Ltd., West Sussex, UK) were added to inhibit proteases. The cells were lysed on ice by ultrasonication (Bandelin Sonopuls sonicator) with six cycles of 30-s on and 60-s rest periods at 45% amplitude. The cell lysate was then centrifuged at 40,000 × *g* for 45 min at 4 °C, and the supernatant was collected.

After centrifugation, the supernatant was loaded immediately onto an Ni-IDA column (Generon, Maidenhead, UK) pre-equilibrated with cold buffer A plus the aforementioned protease inhibitors, using a peristaltic pump (GE Healthcare, Little Chalfont, UK). The column was washed with ∼100 ml of buffer A, and the flow-through was discarded. Proteins were eluted from the column by washing consecutively with increasing concentrations of imidazole (30 mm (250 ml), 60 mm (150 ml), 120 mm (100 ml), and 180 mm (100 ml) in buffer A). Each eluted fraction was analyzed both by UV-visible spectroscopy (250–800 nm), and by SDS-PAGE. Fractions containing relatively pure CYP126A1 (mainly the 80, 120, and 180 mm samples) were pooled and concentrated to ∼100 ml using ultrafiltration with Amicon concentrators (Merck-Millipore) at 4 °C. Thereafter, concentrated protein was dialyzed into 50 mm Tris-HCl (pH 7.2, dialysis buffer) containing 50 mm KCl and 1 mm EDTA to remove excess imidazole. The dialyzed protein was then loaded onto a Q-Sepharose column (10 × 4 cm) pre-equilibrated with the dialysis buffer, and the column was then washed and the protein was eluted with a linear gradient of KCl (50–500 mm) in dialysis buffer using an AKTA purification system (GE Healthcare). Fractions were analyzed by UV-visible spectroscopy and by SDS-PAGE as before. Samples with high *A*_418_/*A*_280_ (or Rz) ratios (≥1) were pooled and concentrated to ∼200 μl by ultrafiltration using a Centriprep 30 concentrator (Merck-Millipore) at 1500 × *g*. The concentrated protein was further dialyzed into 10 mm Tris-HCl (pH 7.5) containing 150 mm NaCl. The protein was then subjected to a final purification step using Sephacryl S-200 size exclusion chromatography on an AKTA purifier. Fractions were again analyzed both by UV-visible spectroscopy and by SDS-PAGE. Fractions with Rz values of ≥1.5 were seen to be pure by SDS-PAGE, and these were pooled and concentrated as before and dialyzed into 50 mm Tris-HCl, pH 7.5, containing 50 mm NaCl and 20% glycerol, and stored at −80 °C until use.

##### LC-MS Studies

Protein stock solutions (∼40 μm) were prepared by dilution of purified CYP126A1 protein (∼500 μm) in 200 mm ammonium acetate buffer, pH 7.0. Samples were buffer-exchanged by size exclusion chromatography using Micro Biospin 6 columns, molecular weight cut-off 6000 (Bio-Rad, Hemel Hempstead, UK). LC-MS was performed on a Xevo G2-S QTof UPLC instrument (Waters, Elstree, UK) coupled to an Acquity UPLC system. Samples were eluted through an Acquity UPLC BEH300 C4 column (1.7 μm, 2.1 × 50 mm) using mobile phases of water with 0.1% (v/v) formic acid (Solvent A) and 95% (v/v) acetonitrile in water, containing 0.01% (v/v) formic acid (Solvent B). A solvent gradient of 95% Solvent A for 5.21 min, 100% Solvent B for 1 min, and 100% Solvent A for 1 min at a flow rate of 0.2 ml min^−1^ over a total run time of 7.29 min was used to elute samples. The electrospray source was operated with a capillary voltage of 2.0 kV and a cone voltage of 40 V. Nitrogen was used as the desolvation gas at a total flow of 850 liters/h. Data acquisition and processing were performed using Micromass MassLynx version 4.1 software with total mass spectra reconstructed from the ion series using the preinstalled MaxEnt algorithm.

##### Nano-ESI Mass Spectrometry

Protein stock solutions (∼20 μm) were prepared as described above for LC-MS. Ligands were prepared as stock solutions in DMSO-*d*_6_ at 0.2–1 mm concentrations. Ligand-protein samples were prepared by diluting protein (10 μl) and ligand stocks (0.5 μl) with ammonium acetate buffer (9.5 μl) to give final concentrations of 10 μm CYP126A1, 5–250 μm ligand, and 2.5% (v/v) DMSO-*d*_6_. Mass spectra were recorded on a Synapt HDMS instrument (Waters UK Ltd., Manchester, UK). Capillaries for nano-ESI MS were purchased from ThermoFisher (Hemel Hempstead, UK). Capillary tips were cut under a stereo microscope to give inner diameters of 1–5 μm and then loaded with 2.5 μl of sample solutions. Given below are the general instrumental conditions used to acquire the reported spectra. However, the parameters were varied over the course of each experiment to observe the strength of protein-ligand complexes under different ionizing strengths. All measurements were carried out in a positive ion mode with an ion source temperature of 20 °C, a capillary voltage of 1.5 kV, a cone voltage of 40 V, and an extraction cone voltage of 4.8 V. All reported spectra were collected with a trap collision energy 30–60 V, transfer collision energy 12–30 V, IMS pressure 5.02 × 10^−1^ millibars, and TOF analyzer pressure 1.17 × 10^−1^ millibars. External calibration of the spectra was achieved using cesium iodide at 100 mg/ml in water. Data acquisition and processing were performed using Micromass MassLynx version 4.1. Mass differences resulting from ligand binding were calculated from the unbound protein peak internal to each spectrum. The unbound protein peak was compared with the relevant 5% (v/v) DMSO-*d*_6_ control spectrum for consistency. Mass differences were divided by the molecular weight of the ligand to calculate binding stoichiometry.

##### UV-visible Absorbance Titrations

Optical titrations to determine *K_d_* values were carried out on a Cary 60 UV-visible spectrophotometer (Varian, UK) according to methods described previously (64–67). All titrations were performed using 1-cm path length quartz cuvettes. For binding studies, the following compounds were used: (i) azole antifungal drugs; (ii) compound hits identified from a high throughput screen (these compounds were identified on the basis of CYP126A1 heme spectral perturbation using a library of ∼40,000 organic compounds); and (iii) a group of small compounds able to bind CYP126A1 heme iron through amine, pyridine, or pyrazole nitrogens.

High throughput compound screening was performed at the Screening Unit at the Leibniz-Institut für Molekulare Pharmakologie (FMP), Berlin, using a 40,000-compound library of organic compounds. In brief, compound stock solutions were prepared in DMSO at 10 mm. Medium throughput pipetting robots (Sciclone 3000, Caliper Life Sciences, Hopkinton, MA) were used to aliquot the library (0.4 μl) into 384-well microtiter plates, and the compounds were diluted in 50 mm Tris-HCl, 50 mm Tris-HCl pH 7.5 buffer (40 μl) containing 10% glycerol to a concentration of 100 μm. The plates were then incubated at 37 °C for 15 min, followed by 5 min of sonication to ensure solubilization of the library compounds and then centrifugation for 10 min to remove any particulate material. Absorbance readings of the plates were done using a plate reader (Safire, Tecan, Reading, UK) as a background value as well as for starting reference spectra of the ligand-free CYP126A1 protein. CYP126A1 (10 μl of 10 μm solution) was then pipetted into the plates, and absorbance spectra were recorded between 350 and 460 nm to analyze compound-induced changes in the CYP126A1 heme spectrum. Control data were also generated during the screening process, including following the addition of miconazole and clotrimazole as verified type II ligands for CYP126A1. Background spectra of the compounds and reference spectra of ligand-free CYP126A1 were subtracted from the spectra generated following the additions of the compound library molecules to generate difference spectra. Measurements at each wavelength were averaged and assigned scores based upon the extent of changes in the difference spectra to determine hit compounds. 15 type I and 15 type II compounds were identified as “hits,” and the top ranking compounds were purchased, where available, from ChemDiv (San Diego, CA) or Vitas M (Apeldoorn, The Netherlands).

Compounds were prepared as stock solutions (0.1–100 mm) in DMSO and titrated (in 0.1–0.2-μl aliquots) into 1-ml cuvettes containing either a solution of CYP126A1 (typically 4–6 μm) in 100 mm KP_i_ (pH 7.0) plus 10 mm KCl (buffer B) or buffer alone. DMSO concentrations did not exceed 1% of the final assay volume, and the CYP126A1 absorbance spectrum was not affected by DMSO within this range. Spectra were recorded continuously between 800 and 250 nm at 25 °C. Spectra collected from the buffer control cuvette were subtracted from protein spectra to remove any optical interference from small molecule absorbance. Difference spectra were generated by subtraction of the initial ligand-free protein spectrum from each successive ligand-bound protein spectrum. The maximum change in absorbance for each difference spectrum (Δ*A*_peak_ − Δ*A*_trough_) was then plotted against ligand concentration. Data were fitted using a standard hyperbolic (Michaelis-Menten) function or the Morrison equation for tightly binding ligands in cases where the *K_d_* for the ligand was ≤5 times the CYP126A1 concentration used in the binding assay. The Hill function was used when plots of induced absorbance change *versus* ligand concentration were clearly sigmoidal in character, as described in previous studies (64–67).

##### EPR and MCD Spectroscopy

X-band EPR spectra of ligand-free CYP126A1 (200 μm) as well as CYP126A1 (200 μm) bound to various ligands (typically 500 μm) were recorded using a Bruker ER-300D series electromagnet and a microwave source interfaced with a Bruker EMX control unit, which was fitted with an ESR-9 liquid helium flow cryostat (Oxford Instruments) and a dual-mode microwave cavity from Bruker (ER-4116DM). Spectra were recorded at a temperature of 10 K, a microwave power of 2.08 milliwatts, and an amplitude of 1 millitesla. Samples were prepared in buffer B, with ligands added from concentrated stock solutions in DMSO. MCD spectra were collected for CYP126A1 in the near-UV-visible and near-IR regions, using Jasco J-810 and J-730 circular dichrographs, respectively, and using an Oxford Instruments superconducting solenoid with a 25-mm ambient bore to generate a magnetic field of 6 teslas. A 0.1-cm path length quartz cuvette was used to record near-IR spectra at a sample concentration of 200 μm. UV-visible MCD spectra for CYP126A1 (also at 200 μm) were recorded using a 0.2-cm path length quartz cuvette in 50 mm potassium phosphate in ^2^H_2_O (pH* 7.5) as buffer (where pH* is the apparent pH measured in ^2^H_2_O using a standard glass pH electrode). Data analysis was done using Origin software (OriginLab, Northampton, MA).

##### Redox Potentiometry

The midpoint potential for the CYP126A1 heme Fe^III^/Fe^II^ couple was determined for the substrate-free CYP126A1 and for the P450 bound to a molecule identified through high throughput screening to induce a substantial substrate-like shift in CYP126A1 Fe^III^ heme iron equilibrium toward HS (compound **1**, (*N*-isopropyl-3-(4-nitrophenyl)-*N*-((3-*N*-(*p*-tolyl)-1,2,4-oxadiazol-5-hexyl)methyl)prop-1-en-2-amine)). Spectroelectrochemical titrations were performed in an anaerobic glove box (Belle Technology) under a nitrogen gas atmosphere. All solutions were degassed under vacuum with nitrogen before use in the glove box. Oxygen levels were maintained at <5 ppm. The concentrated CYP126A1 protein samples were passed through a Sephadex G25 column (1 × 20 cm) (10DG column, Bio-Rad) immediately upon admission to the glove box to remove traces of oxygen. This column was pre-equilibrated, and proteins were buffer-exchanged into anaerobic 100 mm potassium phosphate, pH 7.0, plus 10% glycerol to stabilize the protein from aggregation during the titration. The CYP126A1 solutions (∼10–15 μm enzyme in 5 ml of buffer) were titrated electrochemically according to the method of Dutton (68, 69), using sodium dithionite as reductant and potassium ferricyanide as oxidant. For compound **1**-bound CYP126A1, the compound was added until no further induction of HS heme iron accumulation was observed. Mediators were added to expedite electronic equilibration in the system (2 μm phenazine methosulfate, 5 μm 2-hydroxy-1,4-naphthoquinone, 1 μm methyl viologen, and 1 μm benzyl viologen) and to mediate in the range between approximately +100 and −480 mV *versus* the NHE (69). 10–15 min were allowed to elapse between each addition of reductant/oxidant to ensure equilibration and stabilization of the electrode reading. Spectra (250–750 nm) were recorded using a Cary UV-50 Bio UV-visible scanning spectrophotometer coupled to a fiber optic probe immersed in the CYP126A1 solution. The potential was measured using a SevenEasy S20-K meter (Mettler Toledo, Leicester, UK) coupled to a Calomel electrode (ThermoRussell, Cupar, UK) at 25 °C. The calibration of the electrode was done by using the Fe^III^/Fe^II^ EDTA couple as a standard (+108 mV). The electrode reading was corrected by +244 mV relative to the NHE. Absorbance data at wavelengths reporting on the transition of the heme Soret band between oxidized (Fe^III^) and reduced (Fe^II^) forms of the CYP126A1 were plotted *versus* applied potential, and the data were fitted using the Nernst function in Origin version 8.0 (OriginLab).

##### Analysis of CYP126A1-dependent Oxidation of Substrate-like Molecules from Compound Screening

CYP126A1 activity assays with type I molecules from HTS studies were carried out with 0.5–1 μm CYP126A1, 10–20 μm spinach ferredoxin or *E. coli* flavodoxin, 2.5 μm *E. coli* flavodoxin reductase, and 10 μm screening compound. Turnover reactions were performed in total volumes of 1 ml (or 5 ml for MS fragmentation analysis) in 50 mm KP_i_, 50 mm KCl, pH 7.0. Reactions were initiated by the addition of 1 mm NADPH with a regenerating system (10 mm glucose 6-phosphate and 2 units of glucose-6-phosphate dehydrogenase) and incubated at 30 °C for 30 min. Reactions were terminated by the addition of 2–5 ml of dichloromethane. The organic phase was extracted twice following centrifugation, evaporated, and dissolved in 50:50 acetonitrile/methanol for LC-MS studies. Samples were analyzed on an Agilent 6550 iFunnel Q-TOF LC-MS with a 1290 Infinity LC system. A ZORBAX Eclipse Plus C18 (2.1 × 50 mm; 1.8 μm) Rapid Resolution HT column (Agilent) was used with gradients of 0.1% formic acid to acetonitrile or methanol to resolve products. Fragmentation data analysis was performed with the MassHunter MSC (Molecular Structure Correlator) program (Agilent).

##### Crystallography and CYP126A1 Structure Determination

Crystals of native, ligand-free CYP126A1 were obtained by mixing 1 μl of 20 mg/ml P450 with 1 μl of a reservoir solution containing 38% (v/v) saturated sodium sulfate, 10% (v/v) 1 m Tris-HCl (pH 6.5) and by incubating at 4 °C for 24–48 h. Before data collection, the CYP126A1 crystals were soaked for 5–10 s in a cryoprotectant solution consisting of mother liquor supplemented with 10% polyethylene glycol 200. The imidazole-ligated structure was solved to a resolution of 1.7 Å by molecular replacement using Phaser, giving a unique solution when using PDB entry 1OXA as a starting model against data extending to a resolution of 2.8 Å (70). Refinement and model building were carried out using ArpWarp, Phenix, and COOT (71). No *F*/σ*F* restrictions were applied to the data during refinement, and detailed data and final refinement statistics are given in Table 4.

The crystal structure of the CYP126A1 complex with the azole drug ketoconazole was obtained by co-crystallization. CYP126A1 (20 mg/ml) was mixed with 2 μl of a saturated ketoconazole solution. Sitting drops were prepared by mixing 1 μl of ketoconazole-bound CYP126A1 with 1 μl of mother liquor (40% saturated sodium sulfate, 0.1 m Tris, pH 7.0). These were incubated at 4 °C and formed small needle-shaped crystals. Streak seeding was then used to generate better quality, rod-shaped crystals in 38% saturated sodium sulfate, 0.1 m sodium cacodylate, pH 6.5. Crystals were soaked in cryoprotectant, as above, and flash-cooled in liquid nitrogen for X-ray diffraction experiments.

Crystals of other CYP126A1 complexes were also obtained by co-crystallization after mixing CYP126A1 with these compounds. The CYP126A1-compound **1** complex formed crystals in 0.1 m carboxylic acids, 0.1 m imidazole-MES, pH 6.5, and 30% ethylene glycol-PEG 8000. The CYP126A1-compound **7** complex formed crystals in 0.2 m sodium sulfate, 0.1 m Tris pH 7.5 buffer, and 20% PEG 3350. Following data collection, refinement and model building were carried out using ArpWarp, Refmac, and Coot (71). Detailed data and final refinement statistics are given in Table 4.

##### Materials

Azole anti-fungal drugs were purchased from MP Biomedicals Inc. Novel compounds (compounds **1–9**) were from the compound library of the Screening Facility at the Department of Medicinal Chemistry, Leibniz Institute of Molecular Pharmacology (Berlin, Germany) or sourced from ChemDiv or Vitas M. Bacterial growth medium (2xYT) was from Melford Laboratories (Ipswich, Suffolk, UK). Unless otherwise mentioned, all other reagents were purchased from Sigma-Aldrich and were of the highest grade available.

## Author Contributions

J. T. C., D. V. L., and S. S. expressed and purified CYP126A1 and performed bioinformatics, spectroscopic, redox potentiometry, ligand binding, crystallographic, and other biochemical studies on the enzyme. K. J. M. analyzed CYP126A1 oxidation of substrates. M. E. K. performed nano-ESI experiments and analyzed data. K. J. M., H. M. G., and S. E. J. R. performed EPR experiments and analyzed data. M. R. C. collected and analyzed MCD data. C. W. L. and D. L. collected and analyzed X-ray diffraction data and solved structures. J. P. v. K. directed high throughput screening studies, and B. R. analyzed data. A. W. M. and C. A. directed studies. A. W. M., D. L., C. A., A. G. C., and K. J. M. designed experiments. A. W. M. wrote the paper with contributions from D. L., K. J. M., M. E. K., C. A., and J. T. C. All authors reviewed and edited the manuscript.

## Footnotes

8 The abbreviations used are:

TB: tuberculosis
MDR: multidrug-resistant
Ni-IDA: nickel-iminodiacetic acid
MIC: minimum inhibitory concentration
HS: high spin
LS: low spin
Rz: Reinheitszahl
ESI: electrospray ionization
CT: charge transfer
HTS: high throughput screening
MCD: magnetic circular dichroism
NHE: normal hydrogen electrode
PDB: Protein Data Bank
IPTG: isopropyl β-d-thiogalactopyranoside.
